# Dual role for miR-34a in the control of early progenitor proliferation and commitment in the mammary gland and in breast cancer

**DOI:** 10.1038/s41388-018-0445-3

**Published:** 2018-08-09

**Authors:** Paola Bonetti, Montserrat Climent, Fabiana Panebianco, Chiara Tordonato, Angela Santoro, Matteo Jacopo Marzi, Pier Giuseppe Pelicci, Andrea Ventura, Francesco Nicassio

**Affiliations:** 1 Center for Genomic Science of IIT@SEMM, Istituto Italiano di Tecnologia (IIT), 20139 Milan, Italy; 2 IFOM, the FIRC Institute for Molecular Oncology Foundation, 20139 Milan, Italy; 3 Department of Experimental Oncology, European Institute of Oncology (IEO), 20100 Milan, Italy; 4 Dipartimento di Scienze della Salute, Università degli Studi di Milano, 20100 Milan, Italy; 5 Memorial Sloan-Kettering Cancer Center (MSKCC), New York, USA

**Keywords:** Cancer stem cells, Non-coding RNAs, Cancer stem cells, Non-coding RNAs

## Abstract

The role of the tumour-suppressor miR-34 family in breast physiology and in mammary stem cells (MaSCs) is largely unknown. Here, we revealed that miR-34 family, and miR-34a in particular, is implicated in mammary epithelium homoeostasis. Expression of miR-34a occurs upon luminal commitment and differentiation and serves to inhibit the expansion of the pool of MaSCs and early progenitor cells, likely in a p53-independent fashion. Mutant mice (miR34-KO) and loss-of-function approaches revealed two separate functions of miR-34a, controlling both proliferation and fate commitment in mammary progenitors by modulating several pathways involved in epithelial cell plasticity and luminal-to-basal conversion. In particular, miR-34a acts as endogenous inhibitor of the Wnt/beta-catenin signalling pathway, targeting up to nine upstream regulators at the same time, thus modulating the expansion of the MaSCs/early progenitor pool. These multiple roles of miR-34a are maintained in a model of human breast cancer, in which chronic expression of miR-34a in triple-negative mesenchymal-like cells (enriched in cancer stem cells—CSCs) could promote a luminal-like differentiation programme, restrict the CSC pool, and inhibit tumour propagation. Hence, activation of miR-34a-dependent programmes could provide a therapeutic opportunity for the subset of breast cancers, which are rich in CSCs and respond poorly to conventional therapies.

## Introduction

Breast cancer is a heterogeneous disease with tumour subtypes defined either by histopathology based on hormone receptors (ER+/–, PR+/–, HER 2+/–) or molecularly, using gene expression-based classifier (basal, HER2, luminal A, luminal B, normal-like) [1]. Emerging evidences suggest that a subpopulation of cells with aberrant stem-like properties, called cancer stem cells (CSCs), can account for the biological and molecular heterogeneity of mammary tumours and may contribute to the emergence of therapeutic resistance and disease relapse [2, 3]. Recently, a functional plasticity within different subset of cells emerged, suggesting that CSCs are not a static entity but rather the result of the acquisition of “stemness” properties by tumour cells. Several signalling pathways involved in development and cellular plasticity have been associated to either normal mammary stem cells (MaSCs) or CSCs of the breast, such as the epithelial-to-mesenchymal transition (EMT) programme, Notch, Hedgehog, Wnt/beta-catenin, and p53 [4–7]. In particular, p53 can limit the expansion of the MaSC pool through different mechanisms, as it regulates the polarity of stem cell division by imposing an asymmetric mode of cell division [6] and negatively regulates the EMT programme [8]. Accordingly, re-stabilization of p53 in mammary tumours by pharmacological treatment reduces the number of CSCs and inhibits tumour progression and growth in vivo [6]. The molecular determinants regulated by p53 that are required for the acquisition and the maintenance of stemness traits in normal and tumour cells remain largely unknown.

Recently, microRNAs (miRNAs), an evolutionarily conserved class of small non-coding RNAs (of 18–24 nucleotides), have emerged as pivotal regulators of gene expression and are involved in a variety of cellular processes, including differentiation, growth control, and cell fate determination (reviewed in [9]). miRNAs negatively regulate gene expression at the post-transcriptional level, with each miRNA able to target several mRNA species. It is becoming evident that miRNAs may act as master regulators of the self-renewal and differentiation of stem cells, and their aberrant regulation in tumours has been shown to participate in the emergence and maintenance of CSCs, especially for breast cancers [10–12]. Some miRNAs have been already reported to be under the control of p53, such as miR-145 [13], miR-107 [14], miR-192, miR-215 [15], and the miR-34 family (of -34a, -34b, and -34c). miR-34 is a tumour suppressor miRNA family that has been discovered as a direct downstream component of the p53 network [16]. Indeed, miR-34 family members are involved in the regulation of a variety of cellular processes relevant in cancer, such as cell-cycle, apoptosis, invasion, EMT, differentiation, and stemness [17] and are frequently downregulated or silenced in tumours, including those of the breast [4, 18]. Of note, ectopic expression of miR-34a has been shown to inhibit prostate [19] and colon CSCs [20], making the miR-34 family potential suppressors of CSCs too. However, the physiological role of miR-34 family and its involvement in p53-dependent phenotypes in the mammary gland and in stem cells (MaSCs or CSCs) remains largely obscure. Here, we investigated the physiological roles of miR-34s in the mammary gland using cell models and mutant (miR34-KO) mice and revealed multiple roles for miR-34a in the control of both proliferation and luminal fate commitment of mammary progenitors.

## Results

### miR-34a/b/c expression in mammospheres and MaSCs

**Fig. 1:**
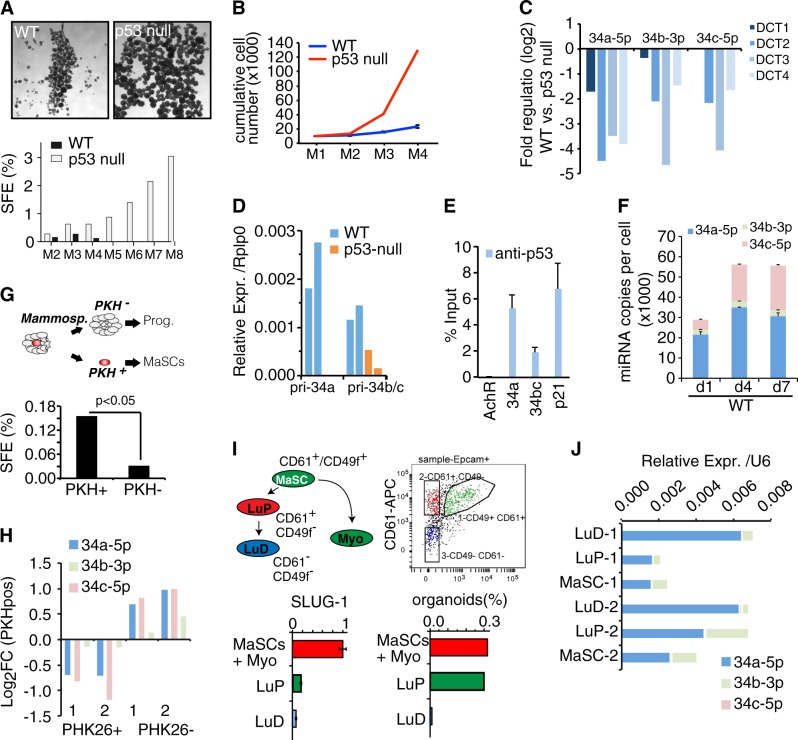
miR-34a/b/c are p53-regulated miRNAs in the mammary gland and increase expression during luminal differentiation. **a**, **b** WT and p53-null mammospheres were analyzed in a serial propagation assay, which measured the sphere-forming efficiency (SFE, %) and the cell number at each passage (**b**). This assay reproduces the “mortal” vs. “immortal” behaviour of MaSCs from WT and p53-null mice. Representative images of mammosphere cultures are shown. Scale bar, 100 μm. One representative experiment (out of four experiments) is shown. **c** RT-qPCR performed on four independent preparations of mammospheres. Data are represented as log2 fold-regulated expression levels of p53 null over WT. **d** Levels of primary transcripts from *miR-34a* and *miR-34b/c* genes were analyzed in WT and p53-null mammospheres by qRT-PCR from two independent experiments. Rplp0 was used for normalization. **e** p53 binding to *miR-34a* and *miR-34b/c* promoters by ChIP-qPCR. Data are reported as percentage of input. Positive control, *p21* promoter; negative control, *AchR* promoter. **f** Absolute levels of miR-34a-5p, miR-34b-3p, and miR-34c-5p (as copies per cell—CPC) in WT M1 mammospheres at day 1, 4, and 7 after plating. The average and SD of triplicate experiments are shown. **g** Scheme showing the PKH26 label-retaining approach and evaluation of sphere-forming efficiency (SFE) on sorted populations. **h** Expression of miR-34c and miR-34a in MaSC that were FACS-sorted using PKH26. Two independent experiments are shown. **i** Scheme showing the mammary epithelial hierarchy and the isolation of subpopulations by FACS profiles of freshly isolated MECs using the CD49f and CD61 markers of MaSCs, luminal progenitors (LuP), differentiated (LuD), and myoepithelial cells (Myo). Bar graphs show mRNA levels of Slug-1, which marks the MaSCs/myoepithelial pool measured by RT-qPCR, and percentage of organoids obtained in 3D Matrigel (which indicates the organogenesis potential typical of MaSC and luminal progenitor cells). **j** Expression of miR-34a and miR-34c measured in the sorted subpopulations described in **i** from WT mice from two independent preparations

We isolated primary mammary epithelial cells from either wild-type (WT) or p53-null mice and grew them as mammospheres, a selective condition for MaSCs. p53-null mice showed an expansion in mammosphere number, highlighting an increased frequency of MaSCs in the mammary epithelium, and an enhanced self-renewal potential (immortal behaviour) (Fig. 1a, b), mimicking the expanded CSC pool typical of the most aggressive (p53-mutated) breast cancers. The three members of the miR-34 family—34a-5p, -34b-3p, and -34c-5p—were all potently downregulated in p53 null mammospheres (Fig. 1c). These miRNAs originate from two distinct genetic loci: one produces the miR-34a transcript, and the other produces the miR-34b and miR-34c primary transcript. Transcription of both genes was dependent on p53 in mammospheres (Fig. 1d) and p53 binding on miRNA promoters was confirmed in mammary epithelial cells (Fig. 1e). In WT cells the expression of miR-34a-5p and miR-34c-5p (which have identical seed sequence and therefore share targets) increased along mammosphere formation, while miR-34b-3p was low expressed and poorly regulated (Fig. 1f). Mammospheres are formed by MaSCs (or early progenitors) with more differentiated cells that physically constitute the sphere. We isolated MaSCs from mammosphere culture through FACS, exploiting a previously validated approach based on labelling (by PKH26) of quiescent stem cells [3, 6] (Fig. 1g). The expression of miR-34s was low in stem cells (PKH26-positive) and much increased in more differentiated cells (PKH26-negative; Fig. 1h). We also measured miRNAs in subpopulations of mammary epithelial cells directly isolated from young (6–8-week-old) virgin mice based on surface markers (as in [21]; Fig. 1i). The expression of miR-34s was low in the subpopulation enriched for MaSCs (CD61+/CD49+; MaSCs and myoepithelial cells) and increased along the luminal differentiation route (luminal progenitors (LuP) and differentiated (LuD) cells; Fig. 1j). In all conditions (primary mammospheres and mammary epithelial cells) miR-34a-5p was the most abundant and regulated miRNA of the family.

### miR-34a expression inhibits self-renewal of MaSCs

**Fig. 2:**
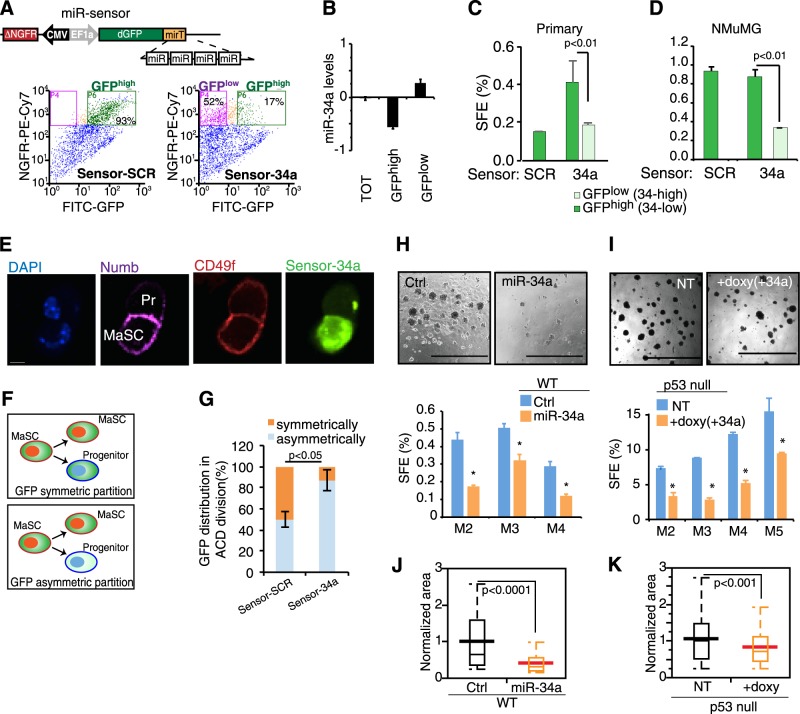
miR-34a expression inhibits self-renewal of MaSCs. **a** Top panel, schematic representation of miR-sensor construct. Lower panel, FACS analysis of WT mammospheres infected with control (sensor-SCR) and miR-34a (-34a) sensor and analyzed by ∆NGFR-PE-Cy7 and GFP intensity (GFP^high^ and GFP^low^ cells). Populations with a fixed ΔNGFR levels were selected by FACS. **b** miR-34a levels measured by RT-qPCR in the total ∆NGFR-PE-Cy7 population (TOT) or from GFP^high^ or GFP^low^-sorted cells. **c**, **d** Sphere-forming efficiency (SFE, %) of mammospheres from GFP^high^ and GFP^low^ using sensor-SCR or -34a in primary mammary epithelial cells (**c**) or in NMuMG cells (**d**). Data represents the average, SD, and *P*-value (Student’s *t*-test) from independent experiments (*n* = 3). **e** Representative image of mammary stem cells (MaSCs) asymmetric cell division (ACD) of cells infected with sensor-34a. GFP expression indicates levels/activity of miR-34a of miR-sensor (GFP^low^ = high miR-34a). The distribution of the fate determinant Numb and the basal marker CD49f were used to monitor ACD and to discriminate MaSCs from progenitor cells (Pr). Bar, 10 µm. **f**, **g** The partition of miR-34a (symmetric versus asymmetric) upon ACD of MaSCs was followed with the GFP intensity of miR-sensor. The plot (**g**) summarizes the results as average, SD, and *P* (Student's *t*-test) from independent experiments (*n* = 3). **h** Mammospheres from WT mice infected with lenti-control (Ctrl) or pCDH-miR-34a-expressing vectors (miR-34a). **i** Mammospheres from p53-null mice infected with the inducible vector pSLIK34a. The serial propagation of mammospheres was used to infer the MaSCs self-renewal potential. Representative images are shown (bar, 2 mm). Plots reporting the average, SD, and *P*-value (Student’s *t*-test) from independent experiments (*n* = 3). Asterisks mark significant differences. *P* < 0.05. **j**, **k** Distribution of the normalized sphere area from **h** and **i**, respectively. **P < 0.01

As MaSCs are supposed to have very low levels of miR-34a as compared more differentiated cells (Fig. 1), we sought to stratify the self-renewal properties of mammary epithelial cells according to the endogenous expression levels of miR-34a. We used a lentiviral miRNA sensor that has a GFP transgene directly responsive to miR-34a (Sensor-34a) due to the presence of four perfect binding sites for miR-34a in its 3′ UTR, plus a second transgene (ΔNGFR, a truncated form of NGFR) for normalization [22] (Fig. 2a). The control sensor (Sensor-SCR) showed a homogenous GFP expression in mammospheres, while two differential populations could be distinguished in mammospheres infected with Sensor-34a: GFP^high^ and GFP^low^ populations, which had low and high levels of miR-34a, respectively (Fig. 2a, b). Interestingly, these two populations showed a remarkably different sphere-forming efficiency (SFE), both in primary mammary cells and in NMuMG cells (*P* < 0.01; Fig. 2c, d), suggesting that miR-34a expression negatively correlates with self-renewal ability. MaSCs are characterized by an asymmetric mode of division, generating one MaSCs and one progenitor per division, thus maintaining the stem cell pool. Using miR sensor, we assessed miR-34a expression during asymmetric division of MaSCs (monitored by Numb and CD49f staining [23]), as previously observed in colon [20]. Indeed, we observed that miR-34a was frequently asymmetrically partitioned (*P* < 0.05; Fig. 2e–g) with MaSCs showing high GFP levels (low miR-34a), thus confirming miR-34a as marker of early progenitor/differentiated cells. Hypothesizing that expression of miR-34a restricts the MaSC pool, we used serial propagation of mammospheres as a surrogate assay to measure MaSC self-renewal. Ectopic expression of miR-34a strongly inhibited the expansion of the MaSC pool in mammospheres from either WT or p53-null mice (Fig. 2h, i). This effect was accompanied by concomitant reduction of cell growth and mammosphere size (Fig. 2j, k). Overall, these data demonstrated that in a normal murine mammary gland, miR-34a acts as a direct downstream p53 target, induced along the commitment/differentiation pathway of LuPs. Expression of miR-34a could restrict the MaSC/early progenitor pool and affect the proliferative potential of early progenitors in a p53-independent fashion.

### Loss of miR-34 affects mammary gland biology

**Fig. 3:**
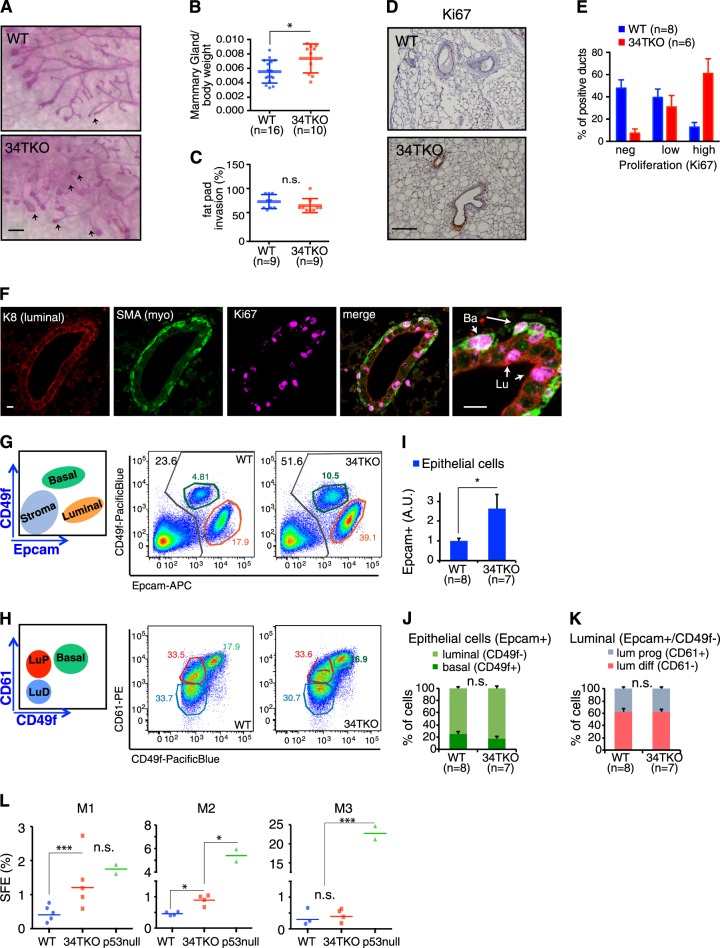
Loss of miR-34 affects mammary gland biology. **a** Whole-mount carmine staining of WT and miR-34TKO (34TKO) mammary glands of 6–8-week-old mice. Arrows mark terminal end buds. Scale bar, 1 mm. **b**, **c** Weight of mammary gland over total body (**b**) and epithelial cells mammary fat pad invasion (**c**) in 6–8-week-old WT and miR-34TKO mice. *P < 0.05 (Welch’s test); n.s. not significant. **d** Immunohistochemical analysis of mammary glands section of the proliferation marker Ki67 and counterstained with haematoxylin. Scale bars, 100 μm. **e** Percentage of mammary ducts positive for Ki67 according to the number of Ki67-positive cells within the ducts in each genotype (WT compared to 34TKO); neg = 0, low = 1–10, high >10-positive cells/duct. **f** Immunofluorescence staining of miR-34TKO mammary gland with Ki67, keratin 8 (K8), and smooth muscle actin (SMA) as luminal and basal/myoepithelial marker, respectively. Scale bar, 10 μm. **g**, **h** Schematic representation and FACS analysis of the WT and miR-34TKO mammary epithelial cells populations identified (**g**). Epcam^high^/CD49f^low^ cells, which represent the luminal population; Epcam^low^/CD49f^high^ cells, which represent the basal/myoepithelial population (**h**). CD49f^high^/CD61^high^ cells, which represent the basal/myoepithelial population; CD49f^low^/CD61^high^, which represent luminal progenitor cells; and CD49f^low^/CD61^low^, which represent luminal differentiated cells. **i** Quantification of FACS analyses of mammary epithelial cells subpopulations in WT and miR-34TKO mice, showing total epithelial cells (Epcam+). **j**, **k** Difference between luminal (CD49f–) and basal (CD49f+) cells (**j**); and separating luminal (Epcam+/CD49f–) cells from progenitors (lum prog, CD61+) or differentiated (lum diff, CD61–) cells (**k**). Data show the average, s.e.m., and *P-*values (Welch’s test) of three independent experiments. *P < 0.05; n.s. not significant. **l** Sphere-forming efficiency (SFE, %) of WT, miR-34TKO (34), and p53-null (p53) mammospheres measured at consecutive passages (M1, M2, M3) from multiple independent preparations. ***P < 0.01, *P < 0.05, n.s. not significant (Welch’s test)

To investigate the role of miR-34a in the physiology of the mammary gland, we took advantage of mice carrying targeted deletion of all miR-34 family members (miR-34TKO; [24]), thus avoiding functional redundancy. Mammary glands of miR-34TKO young (6–9-week-old) virgin mice were significantly bigger than age-matched controls, but no major alterations emerged during development (Fig. 3a–c). In particular, terminal end buds were larger, suggesting an effect on proliferation of early progenitors after loss of all miR-34s. Immunohistological analyses confirmed that miR-34TKO mice had hyperplastic mammary glands, with enhanced proliferation, as shown by increased Ki67 (Fig. 3d, e). Using cytokeratin 8 (K8) as luminal, and smooth muscle actin (SMA) as basal/myoepithelial marker, we found increased proliferation in both compartments, suggesting that miR-34 loss increased overall proliferation, perhaps by affecting early progenitor cells (Fig. 3f). Very similar results were observed also in the mammary gland of p53-null mice (Supplementary Fig. 1a, b). We analyzed surface markers by FACS and observed that the miR-34TKO mammary glands had a significantly higher content of total epithelial cells (Epcam^+^; Fig. 3g, i), in agreement with the histological analyses. The overall distribution of basal compared to luminal cells, and progenitor compared to differentiated cells, was maintained (Fig. 3g, h, j, k). We next analyzed miR-34TKO mammospheres. While we found a significantly higher number of sphere-forming cells in mammospheres from miR-34TKO mice compared to p53-null mice, this effect was restricted to the first passages (Fig. 3l and Supplementary Fig. 1c). These results suggested a transient amplification within the MaSC compartment, which would not be sufficient to acquire the fully expanded and “immortal” behaviour typical of a p53-null background. Accordingly, miR-34TKO mammospheres maintained a luminal profile (CD49f^–^/CD61^+^) similar to WT cells and in contrast to p53-null mammospheres (which acquired a basal phenotype, CD49f^+^/CD61^+^; Supplementary Fig. 1d). Further, the mammary repopulating units did not increase (Supplementary Fig. 1e, f). Taken together, these results highlighted a role for miR-34 in controlling mammary gland development through the expansion of the early progenitor pool. As compared to the effect of p53 loss in the MaSC compartment, miR-34 ablation was not sufficient to recapitulate the expansion in MaSCs number or their basal and “immortal” phenotype.

### miR-34a controls fate commitment and proliferation of progenitor cells by modulating several pathways

**Fig. 4:**
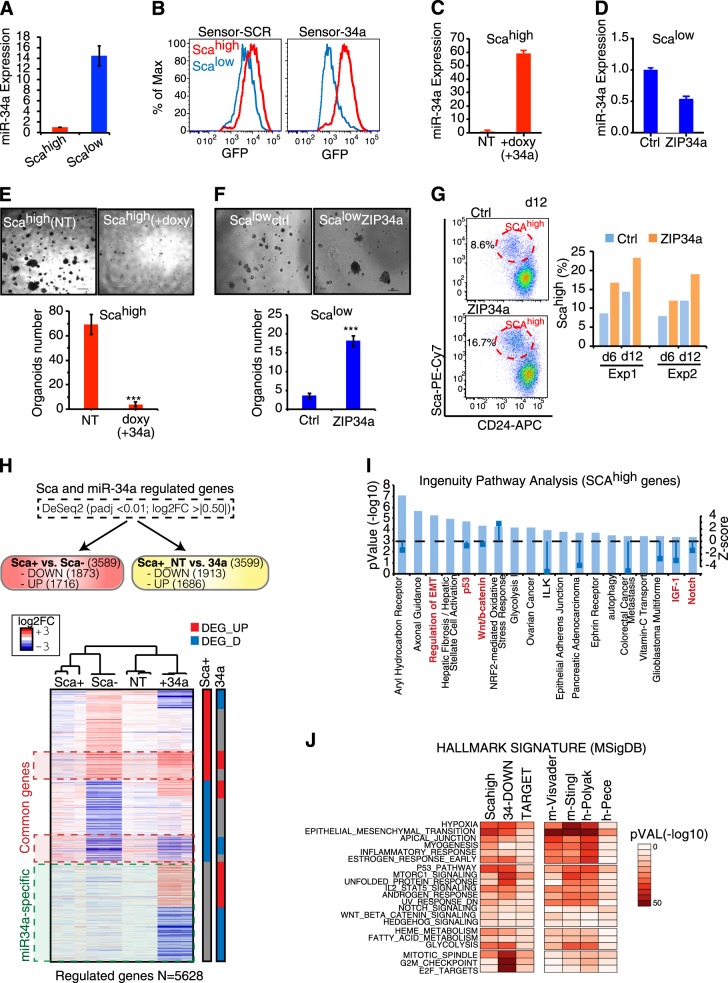
miR-34a controls fate commitment and proliferation of progenitor cells by modulating different pathways. **a** Levels of miR-34a in Sca^high^ and Sca^low^ populations isolated by FACS. Average and SD of three independent experiments are shown. **b** The levels/activity of miR-34a was inferred in the Sca^high^ and Sca^low^ cells by means of GFP intensity with miR-sensor. **c**, **d** The levels of miR-34a were manipulated in Sca^high^ and Sca^low^ populations by inducing overexpression (with pSLIK-miR34a, inducing miRNA with doxyclicline) or knockdown (with miRZIP34a). Graphs show miR-34a expression levels in control compared to treated cells. The average and SD from three independent experiments are shown. **e**, **f** Efficiency of organoids formation in 3D Matrigel from Sca^high^ and Sca^low^ cells upon miR-34a manipulation. One thousand cells were plated. A representative picture is shown. Scale bar, 100 μm. The average, SD, and *P*-values (Student’s *t*-test; ***P < 0.01) from multiple independent experiments (*n* = 3) are shown. **g** Spontaneous conversion of Sca^low^ into Sca^high^ cells measured upon miR-34a knockdown (ZIP34a) by FACS at 6 and 12 days post-infection in two independent experiments. **h** Heatmap of the differentially expressed genes (DEGs) in Sca^high^ (Sca+) vs. Sca^low^ (Sca–) and Sca^high^ upon miR-34a expression (+34a). Each sample was analyzed in triplicate. Red or green dashed lines define the common or miR-34a-specific genes, respectively. **i** A bar plot summarizing the results from the Ingenuity Pathways Analysis (IPA) performed on Sca^high^ DEGs. The most significant enriched pathways with *P-*values (–log_10_, left axis) and *Z*-score (right axis) of activation status are given, and the most relevant pathways for MaSCs/progenitor biology are highlighted. **j** Enrichment of hallmark signatures (MSigDB) calculated for various signatures, including Sca^high^ DEGs (Sca^high^), miR-34a Sca^high^ downregulated genes (34-DOWN), miR-34a predicted targets (TargetScan and miRanda; TARGET), and four additional signatures related to MaSCs/CSCs from the literature. A colour code from light red to dark red marks the significance of the enrichment

CommaDβ cells are a normal murine mammary epithelial cell line composed by two different populations in equilibrium: one expressing high levels of stem cell antigen-1 (Sca-1), which displays properties of mammary stem/progenitor cells (Sca^high^ cells); and the other with low expression of Sca-1, composed by differentiated/luminal cells (Sca^low^ cells) [25]. The two populations showed differential expression of basal and luminal genes, along with different morphogenetic properties (Supplementary Fig. 2a–c). Strikingly, miR-34a was highly expressed and more active in Sca^low^ cells than Sca^high^ cells (Fig. 4a, b and Supplementary Fig. 2d). We next performed functional experiments by modulating miR-34a on either Sca^high^ or Sca^low^ cells using a lentiviral inducible vector (pSliK34a, doxycycline inducible) and a lentiviral miRNA sponge vector (miRZIP-34a), respectively (Fig. 4c, d). Consistently, miR-34a overexpression almost completely inhibited Sca^high^ cells from forming organoids (Fig. 4e), while miRNA inhibition enabled Sca^low^ cells to form such organoids (Fig. 4f). Because CommaDβ cells provided a model for studying the plasticity of mammary progenitors (Supplementary Fig. 2e), we wondered if miR-34a expression limits the spontaneous conversion of the more differentiated Sca^low^ cells into the early progenitor state (Sca^high^). Indeed, miRNA downmodulation in Sca^low^ cells resulted in a consistent increase in the Sca^high^ population (Fig. 4g). To identify pathways regulated by miR-34a in mammary progenitors, we performed transcriptome analysis of the two populations (Sca^high^ vs. Sca^low^) and of Sca^high^ cells after early induction (24 h) of miR-34a (Sca^high^NT vs. Sca^high^34a) (Supplementary Fig. 3a). As expected, Sca^high^ and Sca^low^ cells showed very different gene expression (“Sca-signature”, 3589 genes) and were enriched in pathways involved in fate determination, plasticity, and cancer, such as EMT, p53 signalling, Wnt/beta-catenin, and Notch pathway (Fig. 4h, i and Supplementary Fig. 3b). After induction of miR-34a in Sca^high^ cells, several genes were regulated (“34a-signature”, 3599 genes). Downregulated genes were enriched for miR-34a predicted targets, suggesting a direct transcriptional effect of the miRNA (Fig. 4h and Supplementary Fig. 3c–e). We could distinguish two transcriptional programmes regulated by miR-34a. One composed by genes specific for miR-34a activation (“34a-specific”, 2039 genes) and enriched for pathways controlling cell cycle, checkpoints, E2F, and MYC targets (Supplementary Fig. 3c), which explain the anti-proliferative role of miR-34a in progenitor cells. Another set of genes overlapped with the “Sca-signature” (1560 genes; 44.7%), often with opposite trend of regulation (“common genes”; Fig. 4h), suggesting that miR-34a somehow induces Sca^high^ cells towards a differentiated (Sca^low^) phenotype, and account for miR-34a ability to influence progenitor cell commitment observed in vivo and in vitro (Figs. 3, 4). We exploited gene expression signatures of MaSC/progenitor cells from independent studies [3, 5, 26, 27] and focused on the pathways common in the 34a-signatures and Sca-signatures. Besides EMT, which was the most enriched in all signatures, many signalling pathways relevant for MaSC biology were significantly enriched, including Notch, which is a direct miR-34a target [28], p53, mTORC, and the Wnt/beta-catenin signalling pathway (Fig. 4j). Thus, multiple pathways are under the control of miR-34a in mammary progenitor cells, with a dual role in regulating cell proliferation and commitment towards luminal differentiation.

### miR-34a controls Wnt signalling in mammary epithelial cells

**Fig. 5:**
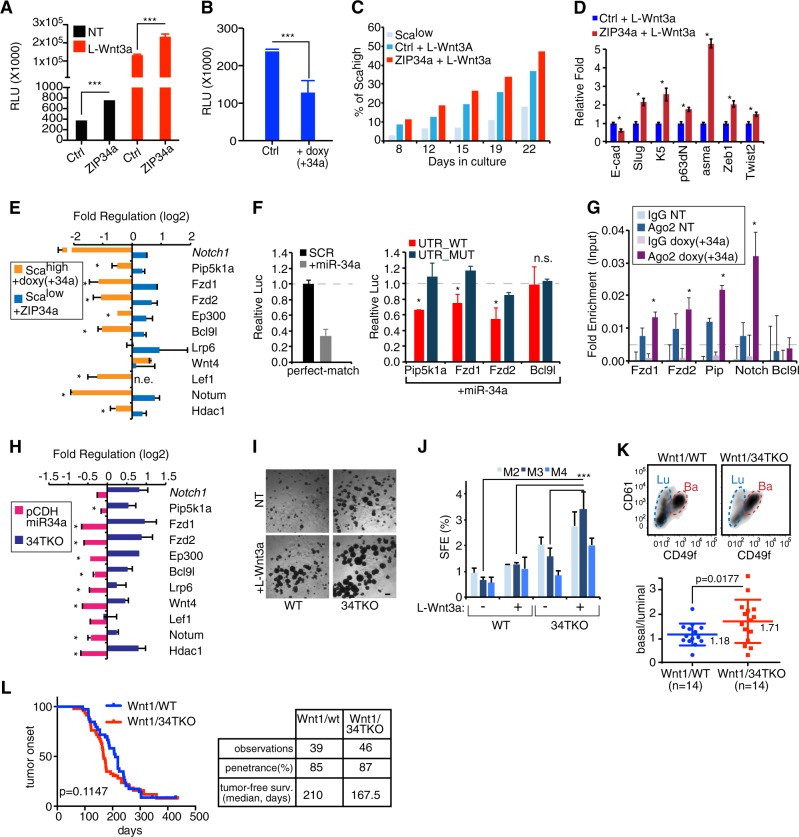
miR-34a controls Wnt signalling in mammary epithelial cells. **a** Sca^low^ cells carrying the Wnt-reporter (7-TCF-luciferase) and infected with control (Ctrl) or miRZIP34a (ZIP34a) vectors were used to measure Wnt signalling activation in basal conditions or after addition of L-Wnt3a ligand (24 h). Data show average and SD (Student’s *t*-test) of technical replicates from one of four experiments. ***P < 0.01. **b** Wnt signalling activation measured by a Wnt-reporter upon miR-34a overexpression (+34) on Sca^high^ cells and ligand stimulation (L-Wnt3a, 24 h). The average and SD of three experiments are shown. *P < 0.05. **c** Conversion of Sca^low^ into Sca^high^ cells by FACS after miR-34a knockdown and L-Wnt3a stimulation. One representative experiment is shown. **d** Panel of luminal and basal markers by RT-qPCR in Sca^low^ cells after miR-34 knockdown and L-Wnt3a stimulation (Student’s *t*-test, *P < 0.05). **e** Expression regulation (log_2_ fold-change) of putative miR-34a targets within the Wnt pathway by RT-qPCR upon miR-34a overexpression (+doxy) in Sca^high^ cells, or a knockdown (ZIP34a) in Sca^low^ cells. The average and SD from three experiments are shown. Asterisks mark significant (Student’s *t*-test) and concordant regulations; n.e. not expressed. *Notch 1* was used as positive control. **f** Luciferase assay of selected miR-34a targets. A perfect match was used as positive control (left panel). For each target, the WT (UTR_WT) and seed-mutated form (UTR_MUT) of their 3′ UTR were used. Data represents average, SD, and *P*-values (Student’s *t*-test; *P < 0.05) from three experiments. **g** RNA immunoprecipitation (RIP) of Ago2-bound RNA in Scahigh cells in basal conditions (NT) or upon miR-34a overexpression (+doxy, 24 h). Shown enrichment over the input in one representative experiment out of two replicates. **h** mRNA expression regulation (average and SD, three experiments) of putative miR-34a targets measured in: (i) miR-34TKO (34TKO) mammospheres compared to the control (WT), and (ii) WT mammospheres after miR-34a overexpression (pCDH miR-34a). Asterisks mark significant (Student’s *t*-test) regulations. **i**, **j** Representative images and serial propagation of mammospheres from WT and miR-34TKO (34TKO) after L-Wnt3a stimulation. Average, SD, and *P-*values (Student’s *t*-test, ***P < 0.001) from three experiments are shown. Scale bar, 200 μm. **k** Distribution of luminal compared to basal cell ratio in the mammary glands of Wnt1/wt and Wnt1/34TKO mice at pre-tumour stage (6-week-old mice). A representative FACS density plot is shown. **l** Kaplan–Meier tumour-free survival curves and *P-*value (Log-rank test). Summary of the penetrance and median survival are reported in table

Wnt signalling pathway has been implicated in different stages of mammary gland development as well as mammary oncogenesis [29–31], and it controls the expansion of the MaSC/early progenitor pool [7]. Indeed, stimulating the Wnt pathway through L-Wnt3a ligands accelerated the spontaneous conversion of Sca^low^ into the undifferentiated Sca^high^ cells (Supplementary Fig. 4a, b). Previously, the Wnt/beta-catenin pathway has been associated with the EMT programme, p53 pathway, and miR-34a in breast cancer [32, 33]. However, it is still unclear whether miR-34a directly controls Wnt signalling in physiological settings and how this interplay affects the biology of the mammary gland. We observed that miR-34a downmodulation in Sca^low^ cells (with high miR-34a levels) significantly increased TCF/LEF transcriptional activity both in the basal state and after L-Wnt3a ligand treatment; conversely, induction of miR-34a expression in Sca^high^ cells (with low miR-34a levels) significantly reduced the Wnt transcriptional activity (Fig. 5a, b and Supplementary Fig. 4c, d). Accordingly, loss of miR-34a cooperated with Wnt stimulation to accelerate the conversion of Sca^low^ cells to Sca^high^ cells and to induce the expression of basal and mesenchymal markers (Fig. 5c, d). We looked for miR-34a direct targets within the Wnt/beta-catenin pathway exploiting target predictions (TargetScan and MiRanda) and gene expression data from CommaDβ subpopulations. We identified 10 genes that acted upstream of the TCF/LEF transcription factors (most of which act as positive regulators) and that had at least one miR-34a responsive element (MRE) in their 3′ UTR, making them potential miR-34a direct targets (Supplementary Table 1). We assessed the expression of these putative targets in CommaDβ subpopulations after miR-34a manipulation: eight targets showed a coherent regulation, as they were downregulated after miR-34a overexpression in Sca^high^ cells, and upregulated after miR-34a downmodulation in Sca^low^ cells (Fig. 5e). We next generated reporter plasmids by cloning the MRE of four potential targets downstream of a luciferase gene. Both Frizzled receptors (*Fzd1* and *Fzd2*) and a phosphatidylinositol kinase (*Pip5k1a*) (which are involved in the transduction of Wnt signals) were confirmed as novel miR-34a direct targets (Fig. 5f). miRNA:Target interaction was further confirmed by Ago2 RNA immunoprecipitation (Fig. 5g and Supplementary Fig. 4e). We further explored the miR-34a/Wnt interaction by primary samples and animal models. First, we derived mammospheres from WT and miR-34TKO mice and re-expressed miR-34a with a lentiviral system (pCDH-34a). Of note, Most of the miR-34a/Wnt targets (9/10) were also coherently regulated in primary mammospheres, being upregulated in miR-34TKO mice and dowenregulated upon miR-34a re-expression (pCDH-34a) (Fig. 5h). Further, stimulating mammary epithelial cells from miR-34TKO mice with L-Wnt3a ligand increased the spheres-forming efficiency over passages, demonstrating that interplay between miR-34 and Wnt controls the expansion of MaSCs/progenitors (Fig. 5i, j and Supplementary Fig. 4f). Deregulation of Wnt pathway in breast tissue leads to mammary oncogenesis [31]. Wnt1 transgenic mice (MMTV-Wnt1) represent a suitable mammary tumour model: the Wnt pathway is constitutively active and sufficient to drive mammary tumorigenesis [34]. Interestingly, pre-tumoural MMTV-Wnt1 mice showed an expanded basal compartment (Supplementary Fig. 4g). This effect was exacerbated in the miR-34TKO genetic background (Wnt1/34TKO) (Fig. 5k), likely as consequence of the increase in the MaSCs/progenitor compartment by Wnt activation [35]. Nevertheless, Wnt1/34TKO mice had only a slight acceleration in mammary tumour onset with similar penetrance as WT mice (85 and 87%, respectively) and no gross change in mammary subpopulations or tumour histology (Fig. 5l and Supplementary Fig. 4h–i). We conclude that a genetic interaction involving miR-34a and Wnt signalling occurs in the mammary gland, with miR-34a inhibiting multiple upstream regulator of the pathway in order to limit early progenitor plasticity. As respect to mammary tumorigenesis, once Wnt signalling is constitutively activated, loss of miR-34s does not accelerate tumour onset any further.

### miR-34a limits self-renewal potential of breast CSCs (BCSC)

**Fig. 6:**
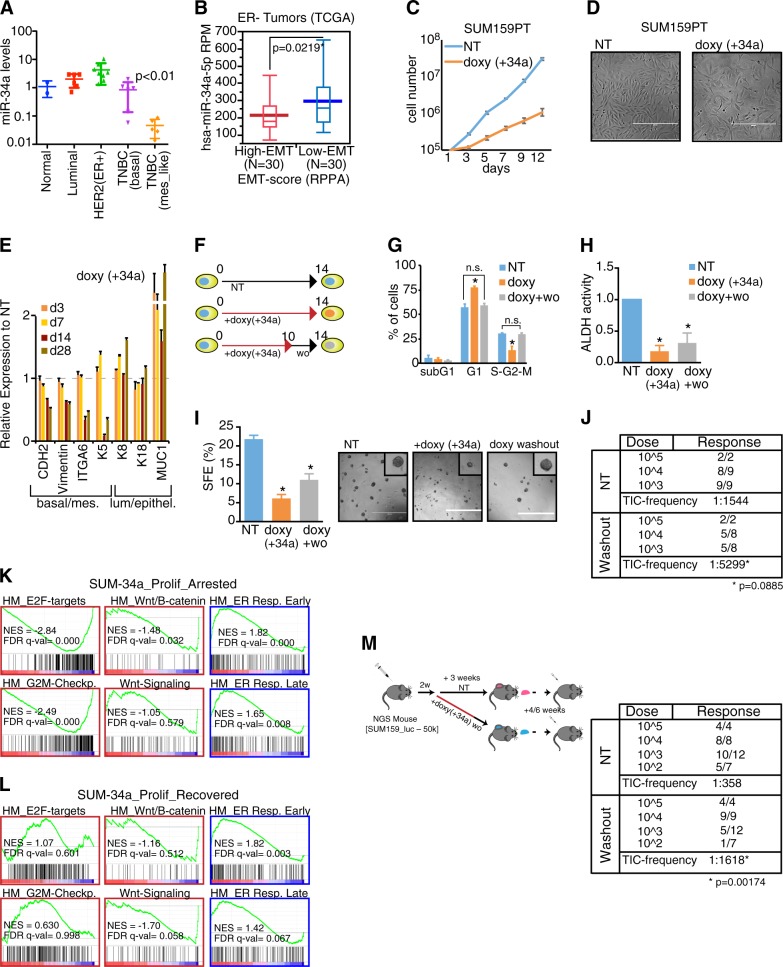
miR-34a limits self-renewal potential of breast cancer stem cells by inducing luminal-like differentiation. **a** Levels of miR-34a in 30 human breast cancer cell lines grouped in five molecular subtypes: normal, luminal, Her2+ER+, triple-negative basal-like (TNBC; (basal)), or triple-negative mesenchymal-like (TNBC; (mes_like)). **b** Levels of miR-34a (normalized reads, RPM) were compared in human ER- breast cancers (TCGA dataset) with different (high vs. low) EMT score. *P*-value (Welch’s test) is reported. **c** Growth curve of SUM159PT cells upon miR-34a expression (doxycycline induced (+doxy)). Average and SD of triplicates are shown. **d** Representative pictures of parental cells (NT) or cells after 7 days of doxycycline treatment are shown. Scale bar, 400 μm. **e** SUM159PT cells were chronically exposed to miR-34a overexpression (+doxy), and the expression of basal/mesenchymal markers or luminal/epithelial genes was measured by RT-qPCR. Expression values were normalized to the control (NT) at each time point in one of the two independent experiments. **f**–**j** SUM159PT cells were exposed to miR-34a expression for 14 or 10 days, with 4 days of washout. Parental cells (NT) or cells treated with miR-34a (with or without washout) were further characterized for cell cycle distribution by FACS (**g**), ALDH activity assay (**h**), sphere-forming efficiency (SFE) (**i**), and tumour xenotransplantation at limiting dilutions (**j**). The average, SD, and *P*-value (Student’s *t*-test; *P < 0.05) from three experiments are shown. **k**, **l** Gene set enrichment analysis (GSEA) correlated the gene expression changes in SUM-34a proliferation-arrested (**i**) or proliferation-recovered (**k**), using different gene sets (all analyses are given in Supplementary Table 4). The enrichment profile (in green), together with the score (NES) and significance (FDR-qval) for each gene set analyzed, is shown. **m** TIC measurement by second passage transplantation at limiting dilution

We hypothesized that the dual function of miR-34a in the early progenitors—of inhibiting proliferation and fostering luminal commitment—could be exploited in pathological conditions, i.e., breast tumour, in which miR-34a is downregulated and is negatively correlated with aggressive phenotype [4, 36]. Indeed, miR-34a levels were low in: (i) primary samples from high-grade, estrogen receptor-negative tumours (G3 ER^–^), which are enriched in BCSC [3], as compared to their matched counterpart (low grade, estrogen receptor-positive; G1 ER) (Supplementary Fig. 5a); (ii) triple-negative tumour cells (TNBC), which have a mesenchymal-like phenotype (a feature associated with BCSC properties) (Fig. 6a and Supplementary Table 2); and (iii) tumours from the Cancer Genome Atlas dataset (TCGA; [37]), which display features of an active EMT programme (high EMT score compared to low EMT score tumours) (Fig. 6b). Thus, miR-34a expression might be detrimental for BCSCs. To verify that re-expression of miR-34a affected BCSC properties, we used a triple-negative mesenchymal-like breast cancer cell line, SUM159PT, that has features of BCSCs and low miR-34a levels [38], infected with the doxycycline-inducible pSlik34a (Fig. 6a and Supplementary Fig. 5b). Expression of miR-34a in this context led to reduced proliferation of the SUM159PT cells (Fig. 6c), as miR-34a can inhibit cancer cell proliferation. Interestingly, chronic activation of miR-34a also induced a flattened morphology with no sign of apoptosis (Fig. 6d). Expression of CD24, a marker of luminal differentiation that is normally low expressed in BCSCs, was significantly increased 10 days after miR-34a induction (Supplementary Fig. 5c). Accordingly, the expression of several mesenchymal/basal markers typical of BCSCs (such as N-cadherin (CDH2), vimentin, ITGA6 (CD49f), and keratin 5 (K5)) was consistently decreased (Fig. 6e), suggesting that miR-34a also inhibited stem cell traits. To evaluate directly this hypothesis, we took advantage of the tuneable system and shut down miR-34a ectopic expression (Supplementary Fig. 5b) to completely recover SUM159PT cells from the cell-cycle block (Fig. 6f, g). Cells that were subjected to chronic miR-34a that were either proliferation-arrested (SUM-34a_PA) or proliferation-recovered (SUM-34a_PR) showed features of having a reduced BCSC pool, as revealed by: (i) the significant reduction of positive cells for ALDH staining, which marks the stem cell pool [39] (Fig. 6h), (ii) the decrease in SFE in the mammosphere assay (Fig. 6i and Supplementary Fig. 6d–e), and (iii) the decrease in tumour-initiating cell (TIC) frequency measured by xenotransplantation experiments at limiting dilution (Fig. 6j). Gene expression analysis confirmed that proliferative pathways (E2F-targets, G2-M checkpoint, *MYC* genes) were inhibited in proliferation-arrested (34a_PA) but not proliferation-recovered (34a_PR) cells (Fig. 6k, l and Supplementary Table 3). Conversely, both cells had a repressed Wnt/beta-catenin signalling and displayed a strong induction of the estrogen response (ER) genes, thus underscoring the ability of chronic miR-34a expression to induce luminal differentiation programme in cancer cells (Fig. 6k, l). Although Wnt targets do not always have a MRE conserved between mouse and human orthologues (summarized in Supplementary Table 1), some targets previously found regulated in mouse progenitors (see Fig. 5) were also regulated in SUM159PT cells, including LEF1 and some FZD receptors. Moreover, a reciprocal regulation with miR-34a levels could be found in either breast cancer cells or primary tumours (Supplementary Fig. 6f), suggesting that Wnt signalling is under control of miR-34a but that the specific targets vary according to the context.

We examined in vivo miR-34a effects by injecting NSG mice with SUM159PT cells and we induced expression of miR-34a after tumour development started (about 2 weeks after transplantation) by doxycycline-containing food (Supplementary Fig. 6a). miR-34a induction significantly reduced tumour growth (e.g., tumour size and weight; Supplementary Fig. 6b, c) and inhibited cancer cell proliferation. We next evaluated the impact of miR-34a expression on the BCSC pool by measuring the frequency of TIC by second passage transplantation at limiting dilution without doxycycline-containing food (Fig. 6m). The treatment with miR-34a significantly reduced the TIC frequency (1:1618 as compared to 1:358; Fig. 6m), suggesting more formally that miR-34a expression inhibited the BCSC pool. As a whole, these results suggest that miR-34a has multiple functions in breast cancer, by acting on both tumour growth and tumour “stemness”.

## Discussion

### miR-34a functions in mammary epithelium by coupling proliferation control and commitment to differentiation

Although miR-34 family was discovered early in miRNA biology as the major component of the p53 tumour suppressor network [16], we only recently have begun to decipher its physiological function, and it may play very different roles in different tissues [24, 40], including osteoclastogenenesis [41], ciliogenesis [42], or cardiac aging [43]. By combining expression studies, mutant mice, and cell models, we have now characterized the roles of miR-34a in the mammary epithelium. Expression analyses indicate that miR-34a is physiologically relevant for mammary epithelium, as it is coherently regulated along mammary gland development (e.g., low or absent in MaSCs and induced upon luminal lineage differentiation). Expression of miR-34a is poorly compatible with MaSC identity. Indeed, MaSCs/early progenitors could be stratified from more differentiated cells in mammosphere culture by simply selecting those cells with low miR-34a expression. Accordingly, expression of miR-34a in MaSCs/early progenitors abolished self-renewal properties as observed by mammospheres. In mutant mice the situation was more complex. An increase in the proliferative ability of both luminal and basal mammary epithelial cells was observed in miR-34 KO mice, similar to p53-null mice. However, the increased number of MaSCs, which is typical in p53-null mice, was not reproduced only by the deletion of miR-34(a/b/c), nor was the “immortal” behaviour or the basal characteristics of primary mammospheres. Hence, while miR-34a is in control of proliferation and mediates, at least in part, p53 anti-proliferative function, it minimally influence MaSC self-renewal under physiological conditions. Besides proliferation, miR-34a also control luminal differentiation. Acute ablation of miR-34a in a mammary progenitor cell line (CommaDβ) elicited the spontaneous conversion of more differentiated cells (Sca^low^) towards an undifferentiated/MaSC-like state (Sca^high^), indicating that miR-34a is implicated in cell commitment and differentiation. Notably, a role for miR-34a in coupling proliferation control and differentiation was previously suggested, based on results from miR-34a overexpression in other non-epithelial progenitors, such as myoblasts [44] and neurons [45]. Therefore, a more general role of such miRNAs in coupling proliferation and differentiation could be envisioned in multiple tissues.

### miR-34a is a natural inhibitor of the Wnt/beta-catenin signalling pathway

Using genome-wide analyses, we identified different pathways that are regulated by miR-34a in mammary progenitor cells. We focused on Wnt/beta-catenin signalling, as it proved to be critically involved in the identity and maintenance of stem cells, both in the normal and in the cancer mammary gland [35]. We showed that miR-34a negatively regulates Wnt signalling in progenitor cells by controlling the expression of multiple genes within the pathway. Up to nine different upstream regulators of the pathway were coherently responsive to modulation (gain or loss) of miR-34a levels either in vitro or in vivo, revealing three novel direct miR-34a targets (Fzd1, Fzd2, and Pip5K1a). An interplay between the Wnt signalling pathway and miR-34a was previously characterized in breast cancer [32, 33] and highlighted the existence of different miR-34 targets within the pathway. Our systematic analysis of such targets using mouse normal cells (CommaDB, primary MECs) and human cancer cells (SUM159PT) (summarized in Supplementary Table 1) revealed that some targets are specific for mouse, often due to low sequence conservation of responsive regions (MREs). However, a significant number of genes in the pathway were indeed regulated, suggesting that, although the gene identities of the targets may differ between species, the function of the miR-34 gene in Wnt signalling inhibition has been conserved. Notably, we showed that Wnt inhibition is not an artefact of the ectopic expression of the miRNA, as it also occurred in a physiological context (e.g., in primary cells, in vivo, loss-of-function). This finding was corroborated by the functional cooperation of miR-34 loss with ligand stimulation (Wnt3A) observed in SCA^low^/SCA^high^ conversion and mammosphere propagation ability (Fig. 5). We can therefore refer to miR-34a as a natural (e.g., physiologically relevant) inhibitor of Wnt signalling pathway in the breast tissue, an activity that critically contributes in restraining the plasticity of mammary epithelial cells.

### The BCSC pool could be limited by miR-34a

A role for miR-34a as therapeutic targets in replacement therapy has been suggested, due to its proven role in inhibiting cell growth in various type of cancers, including lung, prostate, liver, and breast (reviewed in [36, 46]). We observed that re-expression of miR-34a in breast cancer cells of the most aggressive subtype (claudin-low triple-negative) could inhibit the growth of cancer cells, as would be expected. Unexpectedly, however, chronic treatment with miR-34a also elicited a luminal-like differentiation programme, thereby limiting the CSC pool. Such activities resemble the physiological function of miR-34a in controlling proliferation and plasticity of progenitor cells in the normal mammary gland, and appear to involve the commitment to luminal differentiation and the inhibition of Wnt signalling pathway. A role for miR-34a expression in limiting CSCs has been previously suggested in prostate [19], colon [20], and gastric cancer [47]. Strikingly, however, we uncoupled the effects on tumour growth from those on the CSC pool. Indeed, miR-34a chronic expression induced a restriction of the CSC pool and global reprogramming towards luminal-like differentiation, which were maintained when the miRNA was no longer provided, a condition that is sufficient to allow cancer cells to re-enter the normal cell cycle (Fig. 6). Remarkably, such effects were executed even in absence of a proficient p53, as indicated by reduced self-renewal of mammospheres from p53-null mice and SUM159PT (which have a mutated p53). Although p53 plays a crucial role in limiting the expansion of the CSC pool in breast [6], p53 restabilization in mammary tumours has had a limited therapeutic efficacy, due to the low frequency of tumours with WT p53 and the genetic pressure towards p53 loss/silencing in human cancer. Thus, the newly identified function of miR-34a in inducing luminal-like differentiation programme, and the fact that it acts downstream and (most likely) independently from p53, might prove to be extremely useful in treating the most aggressive breast cancers with the worst prognosis, which are rich in CSCs [3] and respond poorly to conventional therapies.

## Methods

### Cell culture and reagents

Cells were isolated from collected mammary glands, and mammosphere cultures were established as described previously [6] (see also Supplemental Materials). Comma-Dβ cells were grown in DMEM:F12 medium (Gibco BRL) supplemented with 2% FBS, 2 mM L-glutamine (Gibco BRL), 10 μg ml^–1^ bovine insulin (Sigma), and 5 ng ml^–1^ murine EGF (Sigma) [25]. The SUM159pt cell line (Asterand, Detroit, MI) was cultured in Ham’sF12 medium supplemented with 5% FBS, 2 mM L-glutamine, 5 µg ml^–1^ insulin, 1 µg ml^–1^ hydrocortisone, 10 mM HEPES, and incubated at 37 °C with 10% CO_2_ in a humidified cell-culture incubator. Doxycycline (Sigma-Aldrich) was dissolved in water (10 mg ml^–1^ stock solution) and used at final concentration of 2 µg ml^–1^. Hygromicin B (Invitrogen; 50 mg ml^–1^ stock solution) was used at final concentration of 200 µg ml^–1^. 4-hydroxytamoxifen (4-OHT; Sigma-Aldrich) was used at final concentrations of 200 or 500 nM.

### In vivo studies

The miR-34TKO mouse strain was generated in the A.V. Laboratory [24], and the Trp53 strain was purchased from Jackson Laboratories (stock #002101); both strains are in the C57/BL6J background. The MMTV-Wnt1 transgenic mouse strain [34] is in the FVB background. Immunodeficient NOD.Cg-Prkdc^scid^IL2rg^tm1Wjl^/SzJ mice (NSG, Charles River) were used for transplantation experiments of SUM159PT.

### Statistics

Analyses (Oneway, Contingency) and statistics were produced using JMP 10 (SAS) software, including data distribution and variance analyses between groups. Microsoft Excel was used to generate bar graphs with average and SD of repeated experiments. The number of replicates and the statistical test used are indicated in figure legends. Heatmaps were generated by Java TreeView software (http://jtreeview.sourceforge.net) for Mac OSX.

### Study approval

All animal studies were conducted with the approval of Italian Minister of Health (12/2012 and 762/2015-PR) and were performed in accordance with the Italian law (D.lgs. 26/2014), which enforces Dir. 2010/63/EU (Directive 2010/63/EU of the European Parliament and of the Council of 22 September 2010 on the protection of animals used for scientific purposes).

## Electronic supplementary material

Supplementary Materials
