# Correction: Dual role for miR-34a in the control of early progenitor proliferation and commitment in the mammary gland and in breast cancer

**DOI:** 10.1038/s41388-019-1094-x

**Published:** 2019-11-08

**Authors:** Paola Bonetti, Montserrat Climent, Fabiana Panebianco, Chiara Tordonato, Angela Santoro, Matteo Jacopo Marzi, Pier Giuseppe Pelicci, Andrea Ventura, Francesco Nicassio

**Affiliations:** 1 Center for Genomic Science of IIT@SEMM, Istituto Italiano di Tecnologia (IIT), 20139 Milan, Italy; 2 IFOM, The FIRC Institute for Molecular Oncology Foundation, 20139 Milan, Italy; 3 IEO, European Institute of Oncology IRCCS, Department of Experimental Oncology, Via Adamello 16, 20139 Milan, Italy; 4 Dipartimento di Scienze della Salute, Università degli Studi di Milano, 20100 Milan, Italy; 5 Memorial Sloan-Kettering Cancer Center (MSKCC), New York, NY USA

**Keywords:** Cancer stem cells, Non-coding RNAs, Cancer stem cells, Non-coding RNAs

**Correction to: Oncogene**

10.1038/s41388-018-0445-3

The original version of this Article contained errors in the author affiliations.

Affiliation number 3 incorrectly read ‘Department of Experimental Oncology, European Institute of Oncology (IEO), 20100 Milan, Italy'. It should read ‘IEO, European Institute of Oncology IRCCS, Department of Experimental Oncology, Via Adamello 16, 20139 Milan, Italy’. Affiliation number 4 incorrectly read ‘Dipartimento di Scienze della Salute, Università degli Studi di Milano, 20100 Milan, Italy'. It should read ‘Department of Oncology and Hemato-Oncology, University of Milan, Via Santa Sofia 9, 20142 Milan, Italy’.

