# Supplementary material for: Dual role for miR-34a in the control of early progenitor proliferation and commitment in the mammary gland and in breast cancer
Source: Oncogene. 2018 Aug 9;38(3):360–74. doi: 10.1038/s41388-018-0445-3 (PMC6336680; doi:10.1038/s41388-018-0445-3)
Supplement: Supplementary file 1 — Supplementary Materials [file 41388_2018_445_MOESM1_ESM.pdf]

Supplemental Methods

Cell Culture and reagents- Mammary tissues from WT or p53 null mice were mechanically dissociated and placed in a digestion medium: DMEM supplemented with 200 U/ml collagenase (Sigma) and 100 U/ml hyaluronidase (Sigma) for 3 hours at 37°C. In order to obtain primary mammospheres, cells were plated onto ultra-low attachment plates (Falcon) at a density of 50,000 viable cell/ml (to obtain primary mammospheres) in a serum-free mammary epithelial basal medium (MEBM, BioWhittaker), supplemented with 5 μg/ml insulin, 0.5 μg/ml hydrocortisone, 2% B27 (Invitrogen), 20 ng/ml EGF and bFGF (BD Biosciences), and 4 μg/ml heparin (Sigma). Mammospheres were collected after 7 days and mechanically dissociated using a fire-polished pipette. For serial passage experiments, 1,000-5,000 cells from disaggregated primary-mammospheres were plated in ultra-low adherent 24-well plates in presence of methylcellulose (MethoCult ™ SF M3236, Stem Cell Technologies) diluted 1:1 in MEBM complemented with cytokines. PKH26 (Sigma-Aldrich) staining was performed on mammospheres, as described (1).

For cell cycle analysis, SUM159PT were fixed in EtOH 70%, stained overnight with 50μg/mL Propidium Iodide and 250μg/mL RNaseA at 4°C, acquired using MACSQuant Flow cytometer (Milteny) and analyzed by FlowJo software (TreeStar). For suspension mammosphere culture, SUM159PT were plated in low-adherent 150 mm dishes (pre-coated with poly-HEMA, Sigma Aldrich) at density of 1000 cells/ml in serum-free mammary epithelial medium (MEBM, Lonza) supplemented with 5μg/ml insulin, 0,5 μg/ml hydrocortisone, 2% B27 (Invitrogen), 20 ng/ml EGF and human bFGF and 4 μg/ml heparin. For serial propagation, mammospheres were collected after 7 days of culture, enzymatically dissociated with trypsin-EDTA (0.025%) and plated at the same density for successive generations. For clonal mammospheres culture, 500 cells were plated in ultra-low adherent 24-well plates in presence of methylcellulose diluted 1:1 to stem medium (with 2x of growth factors).

For 3D Matrigel culture, 24-well plates were pre-coated with Matrigel (BD Biosciences, 120 uL/well) 15 minutes at 37°C. Cells were then seeded at 5,000 cells/well density and incubated for 14 days, medium was replenished every 2-3 days. L-Wnt3a ligand was produced using mouse fibroblast transfected with Wnt3a expressing vector as described (2).

Transplantation experiments. SUM159PT, previously infected with a lentiviral vector encoding luciferase (Addgene, #17477) and with pSlik34a, was injected in 6–8-week-old NSG female mice. For miR34a overexpression, mice were fed with doxycycline–containing food (625 $mg\,kg^{-1}$). Tumour growth was monitored weekly using caliper measurements. For measurements using IVIS Illumina System (Caliper Life Sciences), mice were injected by i.p. with D-luciferin (150 $mg\,kg^{-1}$) and imaged 15 min after injection. Animals were euthanized when the tumours were approximately 0.5–1 cm in the largest diameter. For all transplantation procedures, mice were anaesthetized with 2.5% avertin in PBS (100% avertin: 10 g of tribromoethanol in 10 ml of tertamyl alcohol [Sigma]). Cells were resuspended at the appropriate cell density in 14 μL PBS, 6 μL Matrigel® (BD Biosciences). For second passage transplantation assay, tumour cells were re-transplanted into NSG mice at limiting dilution, with no further treatment with doxy. TIC frequency was calculated with the Extreme Limiting Dilution Analysis (ELDA) web tool.

ALDH assay. ALDH activity was measured using Aldefluor Kit (Stem Cell Technologies #01700). Briefly, SUM159pPT were incubated for 45 min at 37°C with enzyme substrate (ALDEFLUOR reagent) or with an enzyme inhibitor (DEAB reagent), and cells were immediately FACS analyzed using MACSQuant Flow cytometer (Milteny).

Wnt-Reporter activity. Sca^{low} or Sca^{high} cells transduced with Wnt reporter vector (7TF) were treated with L-Wnt3a and collected after 24 hours. For luciferase activity cells were lysed and assayed in multiple wells using the Dual-Luciferase Reporter Assay System (Promega) and a PHERAstar FS plate reader.

3′ UTR luciferase binding assays. HEK293T cells were seeded at 10 000 cells per well in 96-well plate. After 24 h, cells were co-transfected with 100 ng of the indicated 3′ UTR reporter construct (pmiR-GLO; Promega) and 40 nM of SCR or miRNA-34a mimics (QIAGEN) using Lipofectamine 3000 (1 µl/well; Invitrogen), according to the manufacturer's instructions. After 24 h, cells were lysed and assayed in quadruplicate wells for Firefly and Renilla luciferase activity using the Dual-Luciferase Reporter Assay System (Promega) and a Pherastar Plus plate reader. Firefly luciferase activity was normalized to Renilla luciferase activity for each transfected well as follows: [Firefly(miR)/Renilla(miR)]/[Firefly(SCR)/Renilla(SCR)]. Data are representative of four independent experiments.

Plasmids, virus production and infection

Plasmids- Lentivirus for microRNA-34a precursors clone was purchased from System Biosciences (PMIRH34aPA-1) and the following sequence of precursor microRNA-34a was subcloned into the doxycycline-inducible pSLIK-Hygro (Addgene, #125737) vector:

GCCCTTATGGAGTCTTGCTAGTTGCCTGGGCTGGTCTTGAACTCCTGGCCTGAAGCGATCCTCCCACCTCG
GCCTCCTGCATCCTTTCTTTCCTCCCCACATTTCCTTCTTATCAACAGGTGCTGGGGAGAGGCAGGACAGG
CCTGTCCCCCGAGTCCCCTCCGGATGCCGTGGACCGGCCAGCTGTGAGTGTTTCTTTGGCAGTGTCTTAGC
TGGTTGTTGTGAGCAATAGTAAGGAAGCAATCAGCAAGTATACTGCCCTAGAAGTGCTGCACGTTGTGGG
GCCCAAGAGGGAAGATGAAGCGAGAGATGCCCAGACCAGTGGGAGACGCCAGGACTTCGGAAGCTCTTC
TGCGCCACGGTGGGTGGTGAGGGCGGCTGGGAAAGTGAGCTCCAGGGCCCCAGGAGCAGCCTGCTCGTG
GGTGCGGAAGGAAAAAGGCACAGGGGCTTGGTGTGGGCGGCTTTTGGCTGGGAGAAGTTTGCACGTA.

Lentiviral miR-34a sensor was generated in Naldini's laboratory (3). Lentiviral Psicoshp53 and control were generated by A. Ventura (4). Lentiviral miRZIP34a and control were purchased from System Bioscience (SBI), clone MZIP000-PA-1 for SCR and MZIP34a-PA-1 for miR-34 KD. Retroviral pBabep53ER was generated in Pelicci's lab (5). Wnt reporter plasmid (7TF) was purchased from Addgene (#24306).

Virus production- For lentiviral production, HEK293T cells were transfected in 15cm plate with: 10 µg of pRSV-Rev, 10 µg of pMDLg/pRRE (gag&pol), 10 µg of pMD2.G (VSV-G), and 20 µg of the lentiviral vector, 250 µl of 2M $CaCl_2$, in a final volume of 2 ml TE 0.1X. The mix was added dropwise to 2 ml of 2X HBS, by bubbling, and then the calcium-phosphate precipitates were added to the cells at 70% of confluence. For virus concentration, supernatant was collected 36 h post-transfection, filtered through a 0.45 µm syringe-filter and ultracentrifuged for 2 h at 22000 rpm at 4°C. Viral pellet was resuspended in MEBM medium at 100X concentration. Viral stock was frozen (-80°C) or directly used to infect target cells at ratio: 10-30 µl virus/100,000 target cells in presence of 1µg/mL polybrene.

IHC-Mammary glands were fixed overnight in 4% formaldehyde at room temperature, embedded in paraffin, and sectioned (5µm). Sections were deparaffinized, subjected to antigen retrieval, and then incubated with antibody against Ki67 (rabbit, SP6, ThermoScientific, RM-9106-R7) and detected with MACH1 Universal HRP-Polymer Detection (Biocare Medical). Slides were counterstained with haematoxylin and finally mounted with Eukitt (Kindler GmbH). To assess the extent of the proliferative activity, the number of Ki67-positive cells were counted using 100x microscopic fields (microscope type) centered on mammary epithelial structures.

Immunofluorescence- Formalin-fixed paraffin-embedded mammary gland sections were stained with antibodies against Ki67 (rabbit, SP6, ThermoScientific, RM-9106-R7), K8 (rat, Troma-I, MABT329), SMA (mouse, Sigma-Aldrich, A5228). Images were acquired on a Leica TCS SP2 microscope equipped with a 63X oil-immersion objective lens (HCX Plan-Apochromat 63X NA 1.4 Lbd Bl; Leica).

Flow cytometry. Primary mammary epithelial cells from wild-type (WT) and miR-34TKO mice were stained with antibodies against CD49f (PerCP-Cy5.5, BioLegend; Pacific Blue, GoH3, 313617), CD61 (PE,2C9.G3; eBioscience, 12-0611-81), Epcam (APC, G8.8; eBioscience, 17-5791-80), lineage markers (biotinylated anti-CD45, -CD31, -Ter119 primary antibodies plus eFluor-450-conjugated streptavidin, 13-0451-85;13-0311-85;13-5921-85; ; and anti-CD45, -CD31 or -Ter119 with PE-Cy7; eBioscience 25-0451-82; 25-0311-82;25-5921-82). Cells infected with miR-sensor were stained with antibodies against ΔNGFR (CD271-PeCy7; BD Pharmingen;562122). For Comma-Dβ cell line, cells were stained with antibodies against Sca-1 (with Pe-Cy7, D7; or PE, D7; eBioscience; 25-5981-82), CD24 (APC, M1/69; FITC, 30-F1, eBioscience;17-0242-82). SUM159PT cell lines were stained with antibodies against CD24 (PE, ML5; BD;555428). Cells were sorted at FACSAria II sorter or acquired using MACSQuant Flow cytometer (Milteny) and analysed using FlowJo software (TreeStar).

Evaluation of modality of SC mitotic division-WT mammospheres infected with Sensor-34a were mechanically dissociated and re-plated in mammary epithelial basal medium (MEBM, BioWhittaker). After 36-48 h, cells were recovered and transferred onto poly-D-lysine coated glass slides (Corning) and fixed with 4% paraformaldeyde. Cells were permeabilized with 0,1% Triton X-100 and stained with antibodies against CD49f (GoH3, ebioscience;555734) and Numb monoclonal antibody (Ab21). Confocal microscopy was performed on a Leica TCS SP2 microscope equipped with a 63X oil-immersion objective lens (HCX Plan-Apochromat 63X NA 1.4 Lbd Bl; Leica).

Transplantation Experiments-WT and miR-34TKO mammary cells were transplanted into the cleared fat pad of 3 week-old syngenic female mice. Transplanted mammary glands were collected 12 weeks after transplantation, fixed in a 4% formaldehyde and colored with carmine alum for whole mount staining (DeOme, 1959).

Whole Mount staining-Mammary glands were fixed in Ethanol/Acetic acid (3:1) and stained with carmine alum at room temperature overnight. Fat Pads were then clarified using BABB solution (Benzylalcohol/Benzylbenzoate 1:2). Images were analyzed at stereoscope.

RNA isolation and RT-qPCR Analysis-Total RNA was purified onto RNeasy columns (Qiagen) and treated on-column with DNase (Qiagen). For gene expression, cDNA was retro-transcribed using iSCRIPT reverse transcriptase (Biorad). Real-time RT-PCR was performed with FAST SYBR Green Master Mix (Applied Biosystems). Primer pairs were designed through computer assisted primer design software (Primer3). The complete list of primers used in this study is shown below.

	Primer Forward	Primer Reverse
mSlug1	CTCACCTCGGGAGCATACAG	GACTTACACGCCCCAAGGATG

mp63Δdn	CCTGGAAAACAATGCCCAGAC	GAGGAGCCGTTCTGAATCTGC
mSma	TGATCACCATTGGAAACGAACG	TGGTTTCGTGGA TGCCCGCT
mKrt5	GACTGAGGAGAGGGAGCAGA	TCCAGCTGTCTACGGAGGTT
mKrt14	TGAGAGCCTCAAGGAGGAGC	TCTCCACATTGACGTCTCCAC
mZeb1	GTTCTGCCAACAGTTGGTTT	GCTCAAGACTGTAGTTGATG
mTwist2	CGCTACAGCAAGAAATCGAGC	GCTGAGCTTGTCAGAGGGG
mEsa	GCTCTTCAAAGCCAAGCAGT	GGTCGTAGGGGCTTTCTCTT
mCdh1	CACCTGGAGAGAGGCCATGT	TGGGAAACATGAGCAGCTCT
mKrt8	GCACTCAGGAGAAGGAGCAG	GGCGGAGGTTGTTGATGTAG
mKrt18	CTTGCTGGAGGATGGAGAAG	CTGCCATCCACGATCTTACGG
mRpp0	TTCATTGTGGGAGCAGAC	TTGGTGGTACAAACAGGTATTGA
hCDH2	AGCCAACCTTAACTGAGGAGT	GGCAAGTTGATTGGAGGGATG
hVIMENTIN	GACGCCATCAACACCGAGTT	CTTTGTCGTTGGTTAGCTGGT
hITGA6	ATGCACGCGGATCGAGTTT	TTCCTGCTTCGTATTAACATGCT
hKRT5	AGGAGTTGGACCAGTCAACAT	TGGAGTAGTAGCTTCCACTGC
hKRT8	CAGAAGTCCTACAAGGTGTCCA	CTCTGGTTGACCGTAACTGCG
hKRT18	TCGCAAATACTGTGGACAATGC	GCAGTCGTGTGATATTGGTGT
hMUC1	TGCCGCCGAAAGAACTACG	TGGGGTACTCGCTCATAGGAT
Pri-miR-34a	AGGACTTCGGAAGCTCTTCTG	AACTTCTCCCAGCCAAAAGC
Pri-miR-34b	TGCCATCAAAACAAGGCACA	TCCGAGGGTTAACTTGCACTT
Pri-miR-34c	CTGAGCCTCCTGTGAATCGT	CTAAGGGCTAGCGGTTCCC
hRpp0	TTCATTGTGGGAGCAGAC	CAGCAGTTTCTCCAGAGC

- For miRNA expression, RNA were reverse transcribed by using miScript reverse transcription kit (QIAGEN) and RT-QPCR was performed using miScript SYBR Green PCR master mix (QIAGEN) and specific miScript primer assays.

	Qiagen Assay number
Mm_miR-34a_1	MS00001428
Mm_miR-34b-3p_1	MS00011900
Mm_miR-34c_1	MS00001442
Mm_miR-182_1	MS00001715
Hs_miR-183_1	MS00003633
Mm_miR-9_1	MS00012873
Hs_miR-31_1	MS00003290
Hs_miR-335_1	MS00003976
Hs_SNORD72_11	MS000033719
RNU6-2	MS00033740

RNA seq. Total RNA was extracted with the miRNeasy Mini (Qiagen) and treated on-column with DNase (Qiagen) and 1ug was purified with Ribozero rRNA removal kit (Illumina). Libraries were generated with the TruSeq RNA Library Prep Kit v2 (Illumina). Next, sequencing was performed on an Illumina HiSeq 2000 at 50 bp single-read mode and 50 million read depth (3x). RNA-seq NGS reads were aligned to the mm9 mouse or hg38 human reference genome using the TopHat aligner (version 2.0.6) with default parameters. Differentially expressed genes (DEGs) were identified using the Bioconductor package DESeq2 based on read counts, considering genes whose q value relative to the control is lower than 0.05 and whose maximum expression is higher than RPKM of 1. Data are deposited on GEO, GSE99401.

Chromatin ImmunoPrecipitation (ChIP)- ChIPs were performed as previously described (6). Briefly, cells were fixed in 1% formaldehyde for 10 min and resuspended in SDS buffer (50 mM Tris-HCl pH 8, 0,5% SDS, 100 mM NaCl, 5 mM EDTA, protease and phosphatase inhibitors). Cells were disrupted by sonication with a Branson 250 sonicator, performing 5 cycles of 30 sec 30% amplitude, yielding genomic DNA fragments with a bulk size of 100-400 bp. Samples were immunoprecipitated overnight at 4°C with 10 μg p53 antibody (NCL-p53-CM5p - Novocastra laboratories). DNA was then purified by Qiaquick columns (Qiagen) and amplified by RT-qPCR with FAST SYBR Green Master Mix (Applied Biosystems). The following primers were used:

	Primer Forward	Primer Reverse
miR-34a	CAGCCTGGAGGAGGATCGA	TCCCAAAGCCCCCAATCT

miR-34b-c	GTTGATCCTGCCCACAGTTACTAGA	ATTAAAACATGAGTCTCCCTGGTCTCT
p21	TAGCTTTCTGGCCTTCAGGA	GGGGTCTCTCCATTCA
AchR	AGTGCCCCCTGCTGTCAGT	CCTTTCCTGGTGCCAAGA

AGO2 **RNA immunoprecipitation**

AGO2 RNA immunoprecipitation (AGO2-RIP) experiments were conducted with the Imprint RNA Immunoprecipitation Kit (Sigma-Aldrich), according to manufacturer's instructions. Briefly, cells were treated with doxycycline (2ug/ml) for 24 hours in order to induce miR-34a expression A total of 5 million cells were used for each RIP experiment. Cells were trypsinized, collected in a tube and washed twice with ice cold PBS. Cells were resuspended in 200 µl of lysis buffer [mild lysis, protease inhibitor cocktail (PIC), ribonuclease inhibitor] and incubated on ice 15'. Cell debris was removed by centrifugation at 16,000g, 10 min at 4°C. A fraction (10%) supernatant was collected as 'input' and stored on ice, while the remainder was used for immunoprecipitation. For each RIP, 20 µl of magnetic beads with Protein A were pre-cleared with 200 ul of Wash Buffer on a magnetic stand and, then, mixed with 5 µg of control antibody (rabbit Anti-Rat IgG) or Anti-AGO2 antibody (mouse monoclonal 2A8 clone by Sigma Aldrich - Cat. Num. SAB4800048) at RT with rotation for 30 min. 5 µg rat IgG or monoclonal Anti-AGO2 antibody were added to the magnetic beads. Samples were washed twice with 0.5 mL of wash buffer, before adding cell lysates (supernatant) and incubating samples at 4°C with rotation overnight. Samples were then separated with the magnetic stand, washed five times (0.5 ml wash buffer, each time) and, finally, resuspended in 0.2 ml of wash buffer. RNA extractions from RIP samples were performed with 0.6 ml (1:3 ratio) of TRIzol LS (Life Technologies; Cat. Num 10296-028) followed by purification on micro RNeasy Micro Kit colums.

ELDA Software- Limiting dilution analysis was performed using the Extreme Limiting Dilution Analysis (ELDA) web tool (http://bioinf.wehi.edu.au/software/elda/) (7). ELDA computes a 95% confidence interval for the active cell frequency in each population group and it implements a likelihood ratio test for the acceptance of the single-hit hypothesis (pvalue).

Supplemental References

1. Cicalese A, Bonizzi G, Pasi CE, Faretta M, Ronzoni S, Giulini B, Brisken C, Minucci S, Di Fiore PP, and Pelicci PG. The tumor suppressor p53 regulates polarity of self-renewing divisions in mammary stem cells. *Cell.* 2009;138(6):1083-95.
2. Willert K, Brown JD, Danenberg E, Duncan AW, Weissman IL, Reya T, Yates JR, 3rd, and Nusse R. Wnt proteins are lipid-modified and can act as stem cell growth factors. *Nature.* 2003;423(6938):448-52.
3. Brown BD, Gentner B, Cantore A, Colleoni S, Amendola M, Zingale A, Baccarini A, Lazzari G, Galli C, and Naldini L. Endogenous microRNA can be broadly exploited to regulate transgene expression according to tissue, lineage and differentiation state. *Nat Biotechnol.* 2007;25(12):1457-67.
4. Ventura A, Meissner A, Dillon CP, McManus M, Sharp PA, Van Parijs L, Jaenisch R, and Jacks T. Cre-lox-regulated conditional RNA interference from transgenes. *Proc Natl Acad Sci U S A.* 2004;101(28):10380-5.

5. Insinga A, Monestiroli S, Ronzoni S, Carbone R, Pearson M, Pruneri G, Viale G, Appella E, Pelicci P, and Minucci S. Impairment of p53 acetylation, stability and function by an oncogenic transcription factor. *EMBO J.* 2004;23(5):1144-54.
6. Tonelli C, Morelli MJ, Bianchi S, Rotta L, Capra T, Sabo A, Campaner S, and Amati B. Genome-wide analysis of p53 transcriptional programs in B cells upon exposure to genotoxic stress in vivo. *Oncotarget.* 2015;6(28):24611-26.
7. Hu Y, and Smyth GK. ELDA: Extreme limiting dilution analysis for comparing depleted and enriched populations in stem cell and other assays. *Journal of Immunological Methods.* 2009;347(1,Äì2):70-8.

Bonetti et al. Suppl. Figure 1

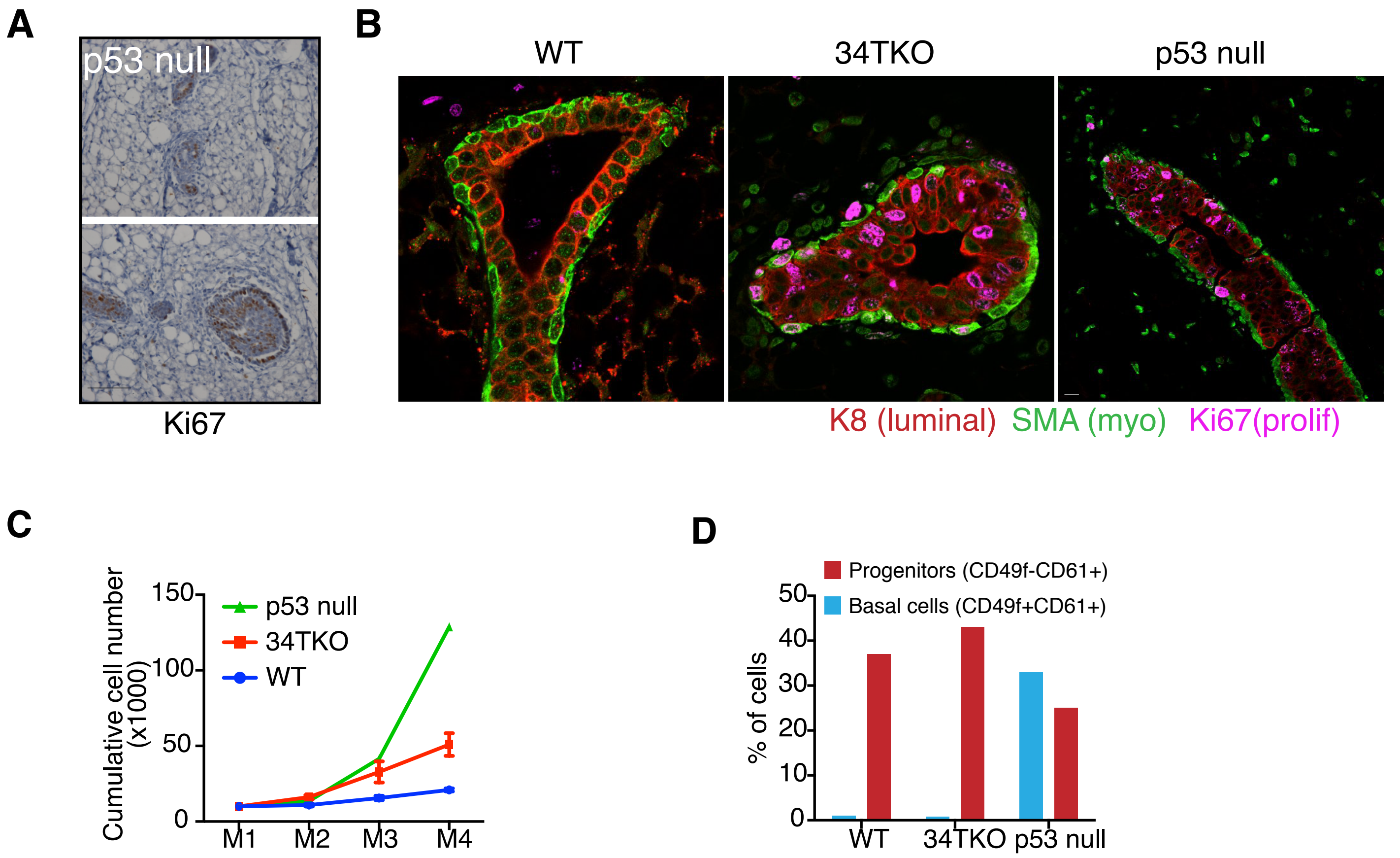

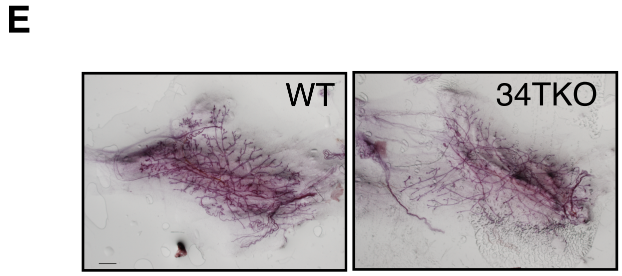

F

	Dose	Response
WT	50000	5/6
	10000	3/8
	1000	0/6
	100	0/6
	MaSC-frequency	1:25977
	(upper/lower)	(12093 - 55799)
34 TKO	50000	6/8
	10000	0/4
	1000	0/5
	100	1/8
	MaSC-frequency	1:37640*
	(upper/lower)	(17096 - 82871)

Supplementary Figure 1 (related to Figure 3). Loss of miR-34 affects mammary gland biology. (a) Immunohistochemical analysis of p53-null mammary glands section by Ki67 (proliferation marker) and counterstained with haematoxylin. Scale bar, 100 µm. (b) Immunofluorescence staining of WT, 34TKO and p53-null mammary gland by Ki67, keratin 8 (K8) and smooth muscle actin (SMA) as luminal and basal/myoepithelial markers, respectively. Scale bar, 10 µm. (c) Growth curve of WT, miR-34TKO and p53-null mammospheres. Cells were plated in triplicate at M1 passage and counted after 7 days. Serial re-plating of mammospheres was performed, and cell numbers were calculated at each passage. The average and SD of triplicate experiments are shown. (d) FACS analysis of WT, miR-34TKO and p53-null mammospheres at M4. Mammospheres were dissociated and stained with antibodies against CD49f and CD61. (e) Representative pictures of carmine-stained whole mount of mammary outgrowths obtained after transplantation of WT and miR-34TKO mammary epithelial cells into syngenic mice. Scale bar, 1 mm. (f) Frequency of the outgrowths upon limiting cell dilutions. MaSC-frequency was calculated with Extreme Limiting Dilution Analysis (ELDA) web tool.

Bonetti et al. Suppl. Figure 2

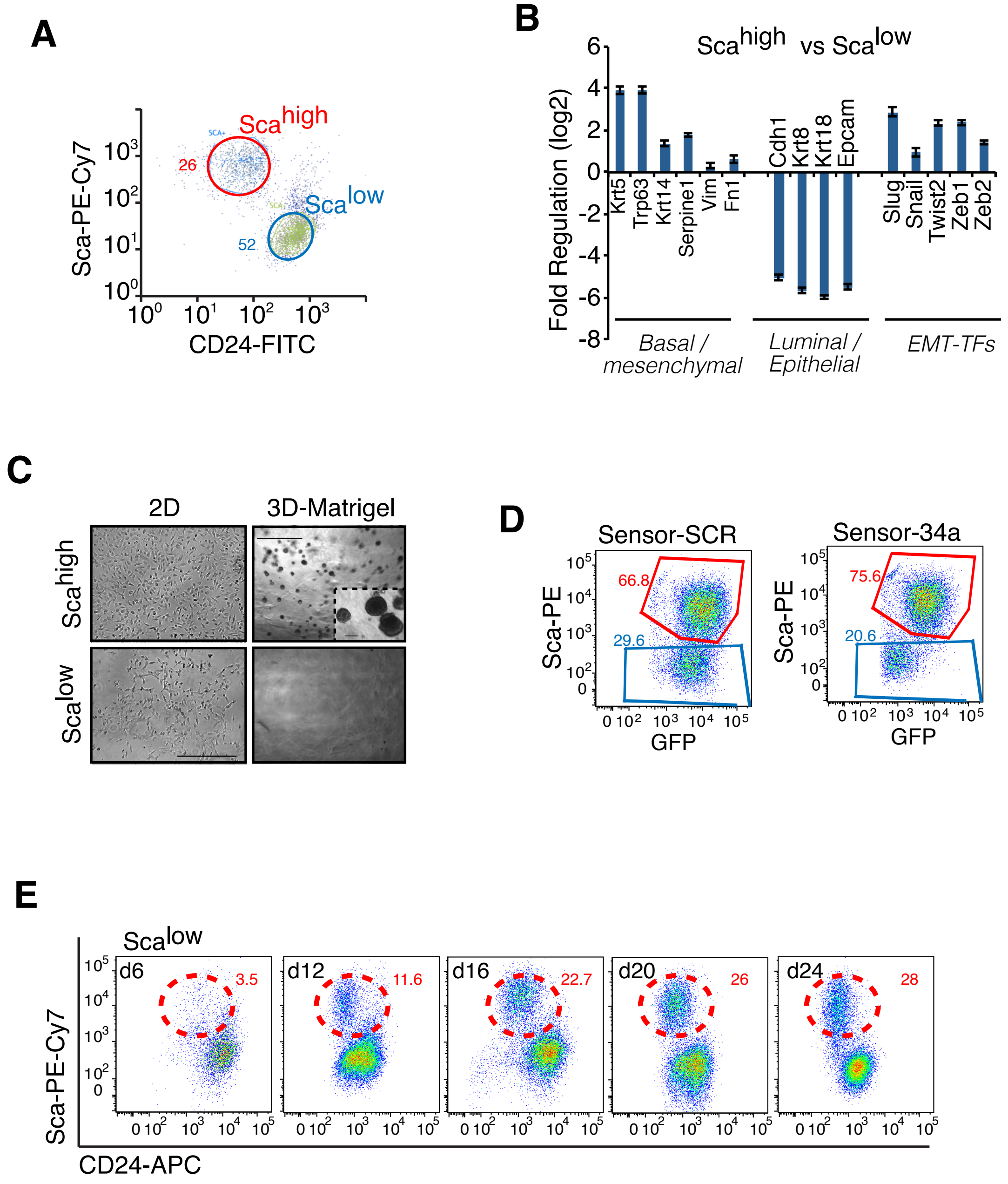

Supplementary Figure 2 (related to Figure 4). MiR34a controls fate commitment and proliferation of progenitor cells by modulating different pathways. (a) Representative dot plot of the CommaDβ cell line stained with antibodies against Sca-1 and CD24, showing the different levels of Sca-1 (Scahigh, Scalow). (b) Levels of basal/mesenchymal, luminal/epithelial and EMT genes in Scahigh compared to Scalow cells (log2 fold-change). The average and SD of triplicate independent experiments are shown. (c) Morphology of the Scahigh and Scalow cells plated in 2D (left) and organoids plated in 3D Matrigel (right) conditions after FACS-sorting. (d) Representative dot plots of CommaDβ infected with sensor-SCR and sensor-34a. Sca-1 and GFP levels of cells that expressed similar amount of ΔNGFR are shown. Scalow cells infected with sensor-34a showed reduced GFP levels. (e) Representative dot plots of FACS-sorted Scalow cells stained with antibodies against Sca-1 and CD24 at different days (from day 6 to day 24) in culture; a significant portion of Scalow cells progressively converts to Sca-high (red-dashed lines) .

Bonetti et al. Suppl. Figure 3

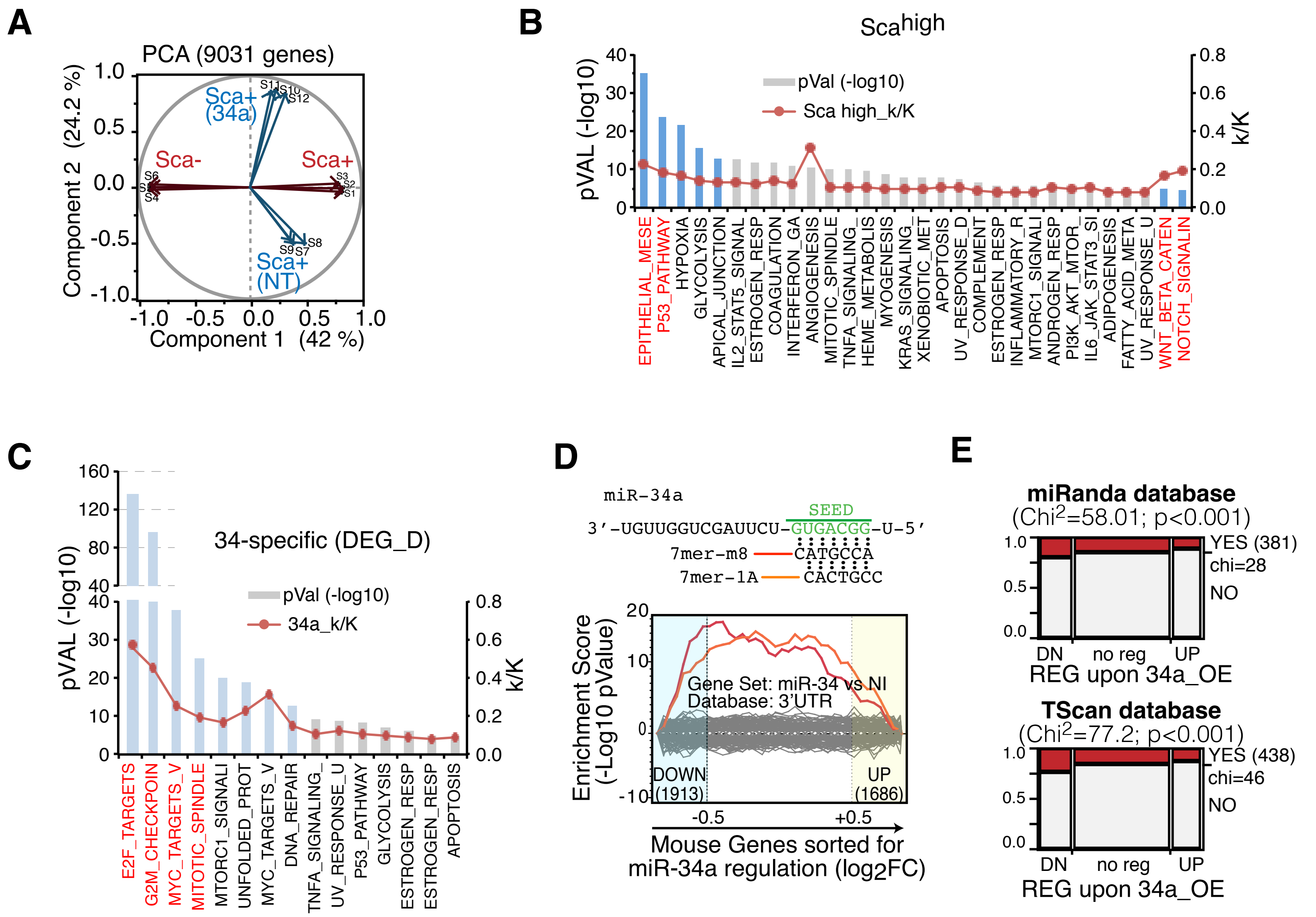

Supplementary Figure 3 (related to Figure 4). MiR34a controls fate commitment and proliferation of progenitor cells by modulating different pathways. (a) Principal Component Analysis (PCA) for individual samples from the RNA-seq analysis of CommaDβ, divided into 4 groups: Sca^{high}, Sca^{low}, Sca^{high} pSlik34a NT and doxycyclin-induced (34a) samples. (b) Histogram summarizing the results of the gene set enrichment analysis (mSigDB) performed with the Hallmark Signature using as input the Sca^{high} DEGs. c) As in (a) but showing the histogram summarizing the results using as input the miR-34a DEGs. The most significant enriched pathways with P values (–log10, left axis) and k/K score (right axis) of activation status are reported. The relevant pathways for MaSCs/progenitor biology and miR-34 regulation that are cited in the main texts are highlighted. (d) Seed enrichment analysis at 3′ UTR sequences of miR-34a expression profile by Sylamer (1). The input list consisted of all expressed genes in the Sca dataset (N = 9031), ordered from the most downregulated to the most upregulated 3′ UTR upon miRNA expression. The two most over-represented motifs correspond to the canonical miR-34a 7mers (7mer-1A and 7mer-m8, as indicated at the top of the panel). (e) Contingency analysis between miR-34a regulated genes [distinguished in upregulated (UP), downregulated (DN), not regulated (no reg)] and targets predicted by either miRanda (top panel) or TargetScan (bottom). Shown the significance of the distribution by the likelihood ratio test (JMP) in the all classes (miRanda Chi2=58.01, $P < 0.001$; TScan Chi2=77.2, $P < 0.001$), or just for downregulated target genes (YES; miRanda Chi2=28, $P < 0.001$; TScan Chi2 = 46, $P < 0.001$) are given.

Bonetti et al. Suppl. Figure 4

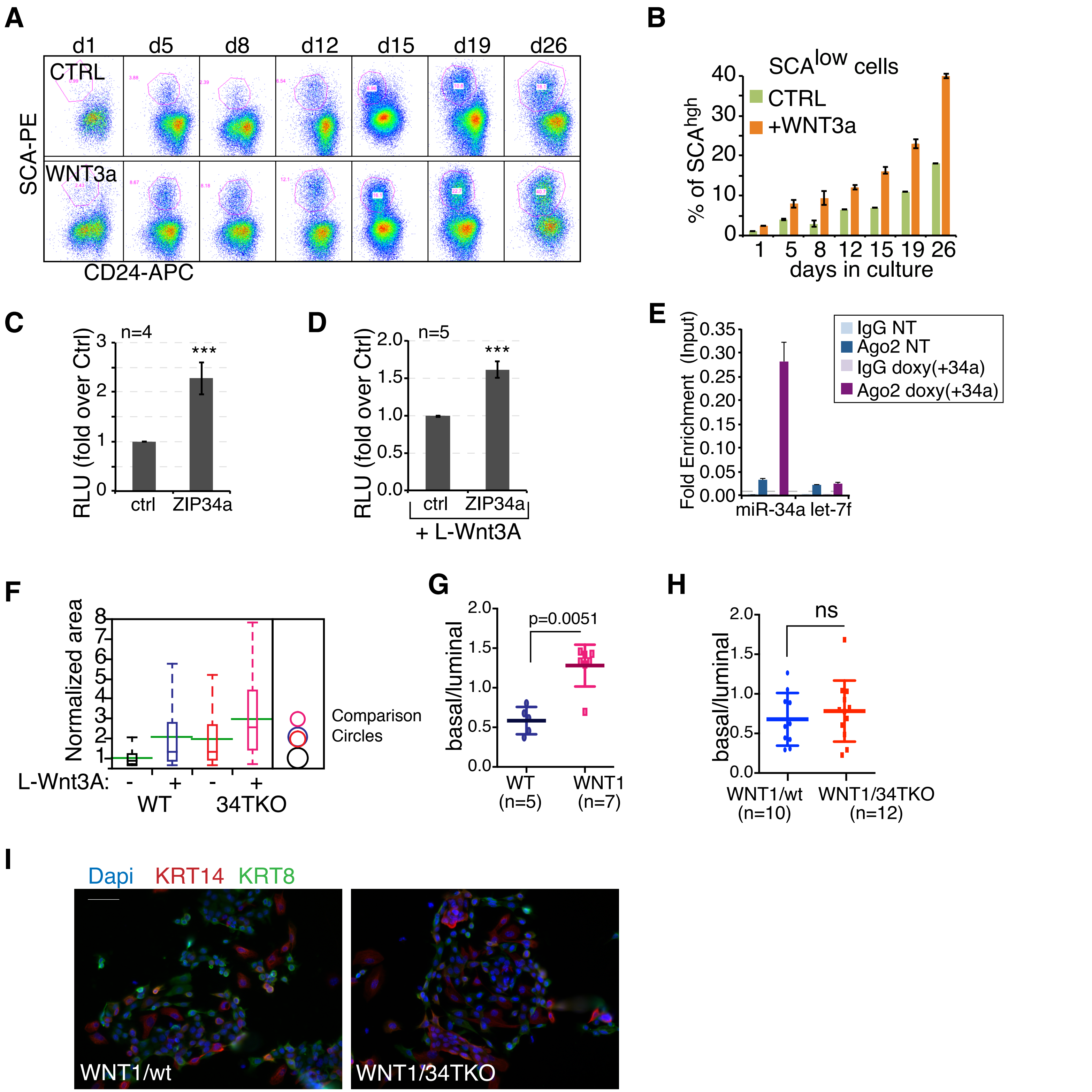

Supplementary Figure 4 (related to Figure 5). MiR-34a controls Wnt signaling in mammary epithelial cells. (a,b) Conversion of Scalow into Scahigh cells observed by FACS upon L-Wnt3a treatment from day 1 to day 26 after treatment. Average and SD of two independent experiments are shown. (c,d) Wnt signaling activation measured by a Wnt-reporter (7-TCF-luciferase) upon miR-34a knockdown (ZIP34a) on Sca^{low} cells in basal conditions (c) or upon ligand stimulation (L-Wnt3a; 24 h) (d). The average and SD of four or five experiments, respectively, are shown. (e) RNA immunoprecipitation (RIP) of Ago2 bound miRNAs in SCA^{high} cells in basal conditions (NT) or upon miR-34a overexpression (+doxy, 24h). (f) Distribution of the normalized sphere area of WT and miR-34TKO (34TKO) mammospheres at M3 passage reported upon L-Wnt3a treatment. (g) Distribution of luminal versus basal cell ratio in the mammary glands of 6–8-week-old WT and MMTV-Wnt1 (Wnt) mice. (h) Distribution of luminal versus basal cell ratio in the tumour sections of Wnt/wt and Wnt/34TKO. Representative images of Wnt/wt and Wnt/34TKO tumour sections stained for keratin (K)14 and K8 antibodies are shown in panel (i). Scale bar, 100 µm.

Bonetti et al. Suppl. Figure 5

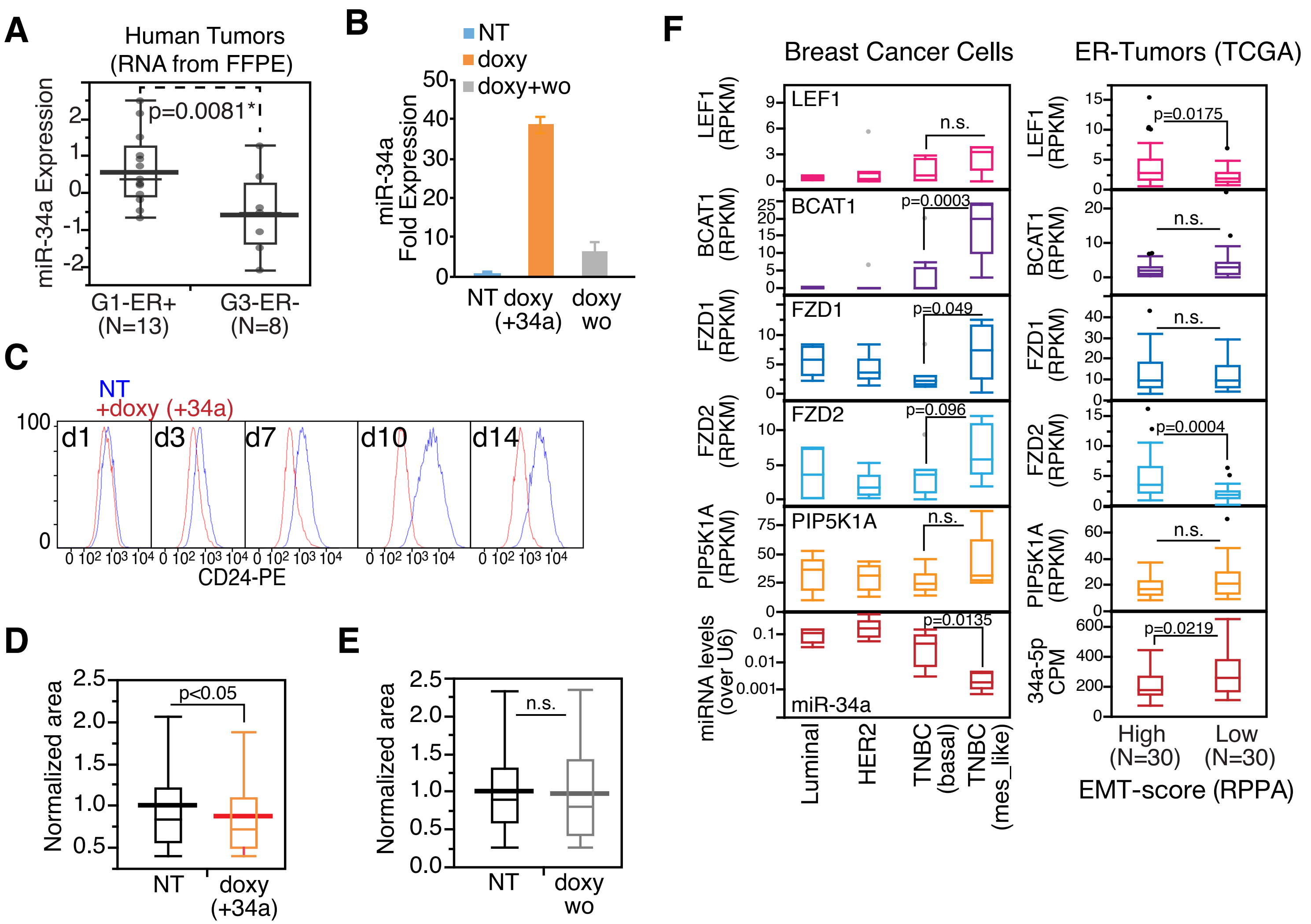

Supplementary Figure 5 – related to Figure 6- miR-34a reduces Cancer Stem Cells in vitro and in vivo (**a**) Levels of miR-34a in a panel of primary tumours (high grade tumours (G3-ER-) vs. low grade tumours (G1-ER+) from IEO hospital. RNA was extracted from FFPE sections and evaluated by RT-qPCR. (**b**) Levels of miR-34a in SUM159PT pSlik34a treated with doxycycline for 14 days and after doxycycline removal (10 days doxy + 4 days washout; (doxy washout)). (**c**) SUM159PT cells were chronically exposed to miR-34a overexpression and the expression of CD24 was measured by FACS analysis at different days. Representative histogram plots at different days of treatment are shown. (**d,e**) Distribution of the normalized sphere area of SUM159PT mammospheres over-expressing miR-34a (+doxy) and after doxycycline removal (doxy washout). (**f**) The expression levels of selected miR-34a targets within the Wnt signaling pathways are shown. Left panel, levels of the genes in human breast cancer cell lines grouped according to molecular subtypes: Luminal, Her2+ER+, Triple negative basal-like [TNBC; (basal)], Triple negative mesenchymal-like [TNBC; (mes_like)]. Shown also the expression of miR-34a in the same samples. A P-value (non-parametric, Dunnet's Methods comparison) between basal and mesenchymal TNBC samples is also shown. Right panel, levels of the genes in human ER- breast cancers (TCGA dataset) with different (high vs. low) EMT-score. P-value (Wilcoxon test) is given. .

Bonetti et al. Suppl/ Figure 6

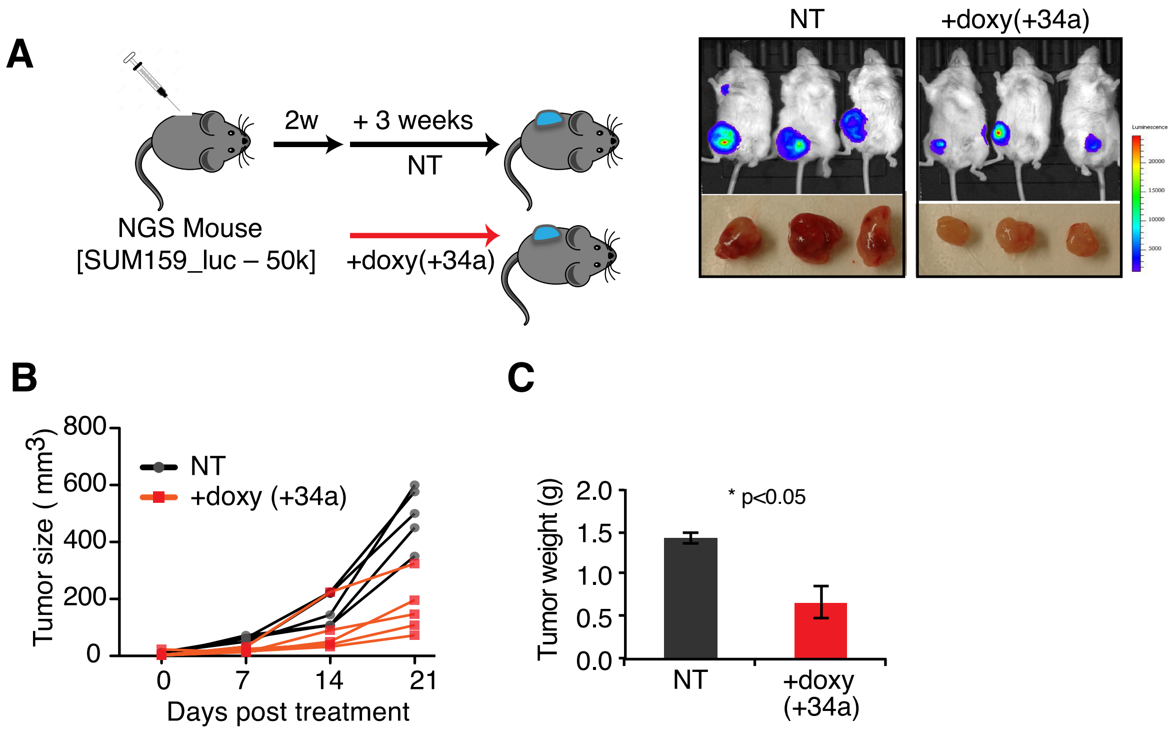

Supplementary Figure 6 – related to Figure 6- miR-34a reduces Cancer Stem Cells in vitro and in vivo (a) SUM159PT cells infected with pSlik34a and LUC were injected into NSG mice and let tumour grow 2 weeks, then fed with doxy or normal food for three weeks. Tumours were monitored using IVIS imaging system. (b,c) miR-34a overexpression (+doxy) significantly reduces tumour growth (b) and tumour weight (c). Tumour size was determined using the formula: $(W^2 \times L) / 2$, where W is width and L is length**.** .

Symbol	Type of reg	Function	Human Ref.seq.	Mouse Ref.seq.	Reference	MRE (mouse)	Validation (CommaDB)	Validation (Mammosph.)	MRE (human)	Validation (SUM+doxy)
WNT1	positive	ligand	NM_005430	NM_053116	Kim ,2011	yes	NE	NE	yes, validated	X
WNT3	positive	ligand	NM _030753	NM_009521	Kim ,2011		NE	NE	yes	NE
WNT4	positive	ligand	NM 030761	NM_009523	new target	yes	NO?	X		
WNT10a	positive	ligand			new target	yes	NO	TBD		
LRP6	positive	receptor	NM _002336	NM_008514	Kim ,2011	yes	NO	NO	yes	NO
FZD1	positive	co-receptor	NM_007197	NM_175284	new target	yes, validated	X	X	yes	NO
FZD2	positive	co-receptor	NM_001466	NM_020510	new target	yes, validated	X	X		NO
NOTUM	negative	antagonist/ligand	NM_178493	NM_175263	new target	yes	X	X	yes	X
IGFBP5	negative	antagonist/receptor	NM_000599	NM_010518	new target	yes	X	X	yes	X
PIP5K1A	positive	kinase	NM_003557	NM_008847	new target	yes, validated	X	X	yes	NO
BCL9L	positive	co-activator	NM_182557	NM_030256	new target	yes	X	X	yes, validated	X
BCAT	positive	transcriptional co-activator	NM_001904	NM_001165902	Kim ,2011,Cha,2014		NO	NO	yes	NO
LEF1	positive	transcription factor	NM _016269	NM_010703	Kim ,2011,Cha,2014	yes	NE	NO?	yes, validated	X
AXIN2	negative	degradation complex	NM _004655	NM_015732	Cha,2014		NO	NO	yes	NO
CD44		target gene	NM_001001392	NM_001039151	Siemens,2011		NO	NO	yes	NO

GROUP	cell line	subtype	dct (U6-miR-34)	log2 FC
1. NORM	HMLE	1. NORM	3.63	1.559950233
1. NORM	MCF-10A	1. NORM	4.91	0.641046067
2. Lum	MCF7	2. Lum	4.07	1.149301154
2. Lum	BT-483	2. Lum	2.74	2.890605639
2. Lum	MDA-MB-175-VII	2. Lum	2.72	2.929028068
2. Lum	HCC1428	2. Lum	4.78	0.704926465
2. Lum	MDA-MB-415	2. Lum	3.71	1.475802846
2. Lum	T-47D	2. Lum	2.72	2.940276953
3. HER2 (lum)	SK-BR-3	3. HER2 (lum)	2.47	3.492255576
3. HER2 (lum)	ZR-75-30	3. HER2 (lum)	2.12	4.435614884
3. HER2 (lum)	MDA-MB-361	3. HER2 (lum)	3.43	1.79599438
3. HER2 (lum)	UACC-812	3. HER2 (lum)	2.82	2.732737449
3. HER2 (lum)	HCC1954	3. HER2 (lum)	2.59	3.218593491
3. HER2 (lum)	HCC2218	3. HER2 (lum)	0.87	10.53394542
3. HER2 (lum)	HCC1419	3. HER2 (lum)	1.52	6.736597054
3. HER2 (lum)	BT474	3. HER2 (lum)	3.82	1.369559398
4. TNBC	HCC1569	4. TNBC	4.14	1.092307807
4. TNBC	BT20	4. TNBC unclassified	6.96	0.154975913
4. TNBC	MCF10A_DCIS	4. TNBC	3.62	1.566414884
4. TNBC	MDA-MB-453	4. TNBC	2.70	2.968563014
4. TNBC	MDA-MB-468	4. TNBC-basal like	4.34	0.953628993
4. TNBC	DU4475	4. TNBC-basal like	3.27	2.003492019
4. TNBC	HCC1143	4. TNBC-basal like	7.10	0.140308079
4. TNBC	SUM149	4. TNBC-basal like	8.39	0.057551103
4. TNBC	HCC1937	4. TNBC-basal like	4.51	0.848826973
4. TNBC	BT549	5. TNBC-mesenchymal-like	8.15	0.067961528
4. TNBC	MDA-MB-231	5. TNBC-mesenchymal-like	10.45	0.013846841
4. TNBC	SUM159	5. TNBC-mesenchymal-like	7.80	0.086802025
4. TNBC	MDA-MB-436	5. TNBC-mesenchymal-like	9.40	0.028672513
4. TNBC	Hs 578T	5. TNBC-mesenchymal-like	9.16	0.03381968

N	GENE SIGNATURE (SUM_34a_Prolif_Arrested)	GS DETAILS	SIZE	ES	NES	NOM p-val	FDR q-val
1	HALLMARK_E2F_TARGETS	Details ...	198	-0.58	-2.84	0	0
2	HALLMARK_G2M_CHECKPOINT	Details ...	196	-0.53	-2.49	0	0
3	HALLMARK_MYC_TARGETS_V2	Details ...	57	-0.64	-2.47	0	0
4	HALLMARK_NOTCH_SIGNALING	Details ...	26	-0.49	-1.6	0.02	0.017
5	HALLMARK_WNT_BETA_CATENIN_SIGNALING	Details ...	31	-0.43	-1.49	0.021	0.033
6	HALLMARK_MYC_TARGETS_V1	Details ...	199	-0.26	-1.27	0	0.137
7	HALLMARK_APICAL_SURFACE	Details ...	19	-0.36	-1.08	0.351	0.412
8	HALLMARK_MITOTIC_SPINDLE	Details ...	187	-0.18	-0.86	0.92	1
9	HALLMARK_SPERMATOGENESIS	Details ...	53	-0.2	-0.76	0.911	1
10	HALLMARK_DNA_REPAIR	Details ...	134	-0.13	-0.58	1	0.994

N	GENE SIGNATURE (SUM_34a_Prolif_REcovered)	GS DETAILS	SIZE	ES	NES	NOM p-val	FDR q-val
1	HALLMARK_BILE_ACID_METABOLISM	Details ...	68	-0.32	-1.28	0.075	0.444
2	HALLMARK_WNT_BETA_CATENIN_SIGNALING	Details ...	31	-0.35	-1.16	0.248	0.512
3	HALLMARK_NOTCH_SIGNALING	Details ...	26	-0.32	-1.04	0.398	0.747
4	HALLMARK_XENOBIOTIC_METABOLISM	Details ...	126	-0.23	-1.02	0.41	0.622
5	HALLMARK_PI3K_AKT_MTOR_SIGNALING	Details ...	84	-0.21	-0.87	0.796	0.92
6	HALLMARK_SPERMATOGENESIS	Details ...	53	-0.19	-0.75	0.881	0.94

FWER p-val	RANK AT MAX	LEADING EDGE
0	3135	tags=62%, list=29%, signal=86%
0	2441	tags=49%, list=23%, signal=62%
0	3127	tags=77%, list=29%, signal=108%
0.051	513	tags=19%, list=5%, signal=20%
0.12	322	tags=16%, list=3%, signal=17%
0.495	7856	tags=99%, list=73%, signal=354%
0.909	1532	tags=37%, list=14%, signal=43%
1	1812	tags=19%, list=17%, signal=23%
1	3155	tags=38%, list=29%, signal=53%
1	1630	tags=12%, list=15%, signal=14%

FWER p-val	RANK AT MAX	LEADING EDGE
0.58	1132	tags=21%, list=10%, signal=23%
0.863	294	tags=13%, list=3%, signal=13%
0.979	1843	tags=38%, list=17%, signal=46%
0.985	1203	tags=16%, list=11%, signal=18%
1	2250	tags=24%, list=21%, signal=30%
1	1484	tags=21%, list=14%, signal=24%
